# Use of Nanoparticles to Prevent Resistance to Antibiotics—Synthesis and Characterization of Gold Nanosystems Based on Tetracycline

**DOI:** 10.3390/pharmaceutics14091941

**Published:** 2022-09-14

**Authors:** Rosa M. Giráldez-Pérez, Elia M. Grueso, Raquel Jiménez-Aguayo, Alfonso Carbonero, Marina González-Bravo, Edyta Kuliszewska, Rafael Prado-Gotor

**Affiliations:** 1 Department of Cell Biology, Physiology and Immunology, Faculty of Sciences, University of Cordoba, 14014 Cordoba, Spain; 2 Department of Physical Chemistry, Faculty of Chemistry, University of Seville, 41012 Seville, Spain; 3 Department of Animal Health, Veterinary Faculty, University of Cordoba, 14014 Cordoba, Spain; 4 Chemtra Company, 47-300 Krapkowize, Opolskie, Poland

**Keywords:** gold nanoparticles, antibiotic resistance, DNA, gemini surfactant, tetracycline

## Abstract

Antimicrobial resistance (AMR) is a serious public health problem worldwide which, according to the World Health Organization (WHO), requires research into new and more effective drugs. In this work, both gold nanoparticles covered with 16-3-16 cationic gemini surfactant (Au@16-3-16) and DNA/tetracycline (DNA/TC) intercalated complexes were prepared to effectively transport tetracycline (TC). Synthesis of the Au@16-3-16 precursor was carried out by using trihydrated gold, adding sodium borohydride as a reducing agent and the gemini surfactant 16-3-16 as stabilizing agent. Circular dichroism and atomic force microscopy techniques were then used to ascertain the optimal R range of the relationship between the concentrations of Au@16-3-16 and the DNA/TC complex (R = C_Au@16-3-16_/C_DNA_) that allow the obtainment of stable and compact nanosystems, these characteristics being fundamental for their use as antibiotic transporters. Stability studies over time were carried out for distinct selected Au@16-3-16 and Au@16-3-16/DNA-TC nanoformulations using the ultraviolet–visible spectrophotometry technique, checking their stability for at least one month. In addition, in order to know the charge and size distribution of the nanocomplexes, DLS and zeta potential measurements were performed in the solution. The results showed that the characterized nanosystems were highly charged, stable and of a reduced size (<100 nm) that allows them to cross bacterial membranes effectively (>1 μm). Once the different physicochemical characteristics of the gold nanosystems were measured, Au@16-3-16 and Au@16-3-16/DNA-TC were tested on *Escherichia coli* and *Staphylococcus aureus* to study their antibacterial properties and internalization capacity in microbes. Differences in the interaction of the precursors and the compacted nanosystems generated were observed in Gram-positive and Gram-negative bacteria, possibly due to membrane damage or electrostatic interaction with internalization by endocytosis. In the internalization experiments, depending on the treatment application time, the greatest bacterial destruction was observed for all nanoformulations explored at 18 h of incubation. Importantly, the results obtained demonstrate that both new nanosystems based on TC and Au@16-3-16 precursors have optimal antimicrobial properties and would be beneficial for use in patients, avoiding possible side effects.

## 1. Introduction

Antimicrobial resistance (AMR) is a serious public health problem worldwide which, according to the World Health Organization (WHO), [1] requires research into new, and more effective drugs. In its Executive Council, the WHO [2] urges member states, among other aspects, to “remain committed at the highest political level to the fight against antimicrobial resistance using a “One Health” approach, and to reduce the burden of morbidity, mortality and disability associated with it; increase efforts to implement the measures and achieve the strategic goals of the Global Action Plan on Antimicrobial Resistance, and take action to address emerging issues” [3]. Thus, in 2017, the WHO made a list that classified the most resistant bacteria to date, dividing them into critical, high and medium priority, according to their danger status [4]. Within the group of bacteria with critical priority are those such as *Acinetobacter baumannii* [5,6], *Pseudomonas aeruginosa* [7] or *Escherichia coli* [8] that have multi-resistance to antibiotics such as cephalosporins or carbapenems, among others, and can cause serious and lethal infections such as sepsis, pneumonia or urinary tract infections. Another example is *Staphylococcus aureus*, which is capable of surviving in adverse conditions and colonizes the skin very easily, penetrating the tissues; the medical conditions that we find most frequently caused by this microorganism are infections of the skin and soft tissues, otitis, osteomyelitis, arthritis, pneumonia and sepsis [9], as it is one of the microbes that generates more nosocomial diseases. Knowledge of the different mechanisms of antimicrobial resistance is essential to be able to propose new strategies that provide a solution to the problem. Thus, among the different mechanisms, the following stand out: those based on the production of inactivating enzymes [10], those based on the modification of the therapeutic target, and those based on the decrease in the intracellular concentration of the antibiotic [10]. Two different pathways generally promote this last mechanism: the action of efflux pumps that expel the antibiotic [11] or the modification of the external bacterial membrane, a mechanism by which the bacteria lose or modify porins, preventing the entry of the antibiotic. An example of this is beta-lactams, hydrophilic antibiotics that pass through porins [12].

This last mechanism, by which the antibiotic fails to reach the interior of the bacterium at a sufficient concentration to be effective, is especially interesting. In fact, this phenomenon could be resolved by implementing a new strategy that achieves, among other characteristics, reduction of the drug concentration required for effective action and adaptation of the environment of the antimicrobial so that it is capable of crossing the bacterial membrane, using nanomaterials of the biomimetic type.

One of the antibiotics that covers a broad spectrum of antimicrobial activity is tetracycline (TC). Discovered in the 1940s, tetracyclines act by inhibiting bacterial ribosomal protein synthesis in prokaryote cells [13]. TC is an antibiotic that helps prevent the multiplication and spread of bacteria. It is used in the treatment of bacterial infections such as those affecting the respiratory tract, skin, eyes, lymphatic system, digestive, reproductive and urinary systems. It is also used against infections caused by lice, ticks, mites, and infected animals [14]. The most used tetracycline in human medicine is doxycycline. Its nucleus consists of a linear tetracyclic structure, which is formed by four fused rings [15]. TC is active against a wide range of both Gram-positive and Gram-negative bacteria. This drug crosses the external bacterial membrane through porins and finally reaches the cytoplasm [15]. The antibiotic generally binds to the ribosomes of bacteria, specifically the 16S ribosomal RNA (rRNA) (small subunit (30S) component); its function is to stop translation [16]. Bacterial resistance is caused by mutations in bacterial rRNA or by the export of TC from the bacterial cell. The latter is mediated by the Tet Repressor (TetR) protein, so when TC is not present, this protein is assembled into DNA and causes the repression of genes that lead to resistance. In contrast, when TC is present, it binds to TetR, so TetR will not bind to bacterial DNA and the expression of the membrane export protein will occur (TetA) [17]. Tetracyclines are mainly administered orally. Absorption will be greater if no food has been previously ingested [18]. It is a toxic antibiotic, so its use is limited; hence the need to control the dose to be administered. In addition, it has the ability to be distributed throughout all body tissues because it is fat-soluble. Resistance to this antibiotic occurs slowly, and it is difficult for it to occur during treatment with this medication; however, there are certain strains that have managed to become resistant to it [18]. Thus, Gram + bacteria reach resistance before Gram −; therefore, the use of this antibiotic against infections by Gram + bacteria is reduced [19].

Nanoparticles are an excellent medium for numerous biological and biomedical applications [20,21]. They can also be used to develop pharmaceutical or physical applications [22]. When a drug is administered with the help of nanoparticles, it reaches the infected tissue, where it is released, thus reducing side effects on healthy tissues [23]. Nanoparticles have unique physical and chemical properties, such as their surface/volume ratio. In addition, surface chemistry is a mechanism used to functionalize nanoparticles by modifying their surface [24]. Nanoparticles are so small (<100 nm) that they can cross bacterial membranes (>1 μm) [25].

In this regard, the improvement of the bactericidal effect achieved by different encapsulation methods in antimicrobials such as ampicillin [26], or clofazimine [27] is noteworthy. Among these, encapsulation using cationic and anionic liposomes is of interest; this method has succeeded in improving some aspects related to the high rate of drug degradation and certain side effects [28]. However, to date, it has not been possible to create preparations of this type that achieve complete encapsulation of the antimicrobial drug, this being approximately 60% at present [29].

Other alternatives described to date resort to the use of nanoantibiotics as antimicrobial agents to replace traditional antibiotics. These nanosystems have some type of antimicrobial activity per se, as is the case with silver, gold, zinc oxide, titanium dioxide or copper nanoparticles [30,31,32,33,34]. Among these, silver nanoparticles (AgNPs), which are used in the coating of surgical materials or the treatment of ulcers, stand out. However, the greatest drawback derived from their use lies in the toxic effects related both to the mechanism of action, based on DNA damage and alteration of the cell membrane, and to their size, though AgNPs of smaller size (10 nm) also have lower toxicity [35]. For this reason, in this study, the use of gold nanoparticles (AuNPs) is proposed as an alternative to AgNPs. AuNPs are frequently used in photothermal therapy and for antifungal use; their mechanism of action is based on their interaction with the bacterial membrane [36] due to their low toxicity [37].

Another example is the use of AgNPs coated with mannose, which is recognized by macrophage membrane receptors and is effective in the treatment of tuberculosis, selectively attacking *Mycobacterium tuberculosis* bacteria [38]. Other current approaches include the use of chitosan nanoparticles (60 nm in size) coated with different antibiotics such as clarithromycin, which are effective as antibacterial agents [39].

Among the most innovative strategies to combat antibiotic resistance problems, the use of nanosystems is remarkable. As a strategy to solve this problem, a new alternative is proposed: the design of new nanosystems containing gold (Au) and coated with biodegradable surfactants (TG) linked to the antibiotic (AT) by means of small DNA chains, Au@TG/DNA-AT. In order to improve and respond to the problems posed, this research has designed and developed a new type of nanosystem with gold as its metallic center, functionalized with non-covalent DNA-TC complexes Au@16-3-16/DNA-TC. For this purpose, the synthesis of gold nanoparticles coated with the hydrophobic 16-3-16 gemini surfactant Au@16-3-16, which will be called the precursor, was taken as a starting point. We then explored the suitable R = C_Au@16-3-16_/C_DNA_ relation of concentrations to guarantee maximum compaction of the DNA-TC complex linked to the cationic Au@16-3-16 precursor. The resulting Au@16-3-16/DNA-TC compacted nanosystems aim to improve the existing selectivity and biocompatibility problems, providing new vehicles for TC transport, which are noteworthy for their low toxicity and great stability (using gold cores instead of silver), as well as reducing the dose used, vectoring the antibiotic and controlling residence time. The Au@16-3-16 precursors and Au@16-3-16/DNA-TC compacted nanosystems obtained were tested at three different concentrations in each case on Gram-positive and Gram-negative bacteria, studying their viability to demonstrate their antimicrobial capacity. Finally, internalization experiments using TEM revealed greater bacterial destruction after 18 h of incubation time with distinct nanoformulations, improving the antimicrobial efficacy of TC.

## 2. Materials and Methods

### 2.1. Materials

All chemicals were of Anal. R. grade and used without further purification. NaBH4 was purchased from Lancaster. Deoxyribonucleic acid sodium salt from calf thymus DNA, TC, hydrogen tetrachloroaurate (III) trihydrate, sodium cacodylate and 3-aminopropyltriethoxilane (APTES) were purchased from Sigma-Aldrich-Merck KGaA (Darmstadt, Germany); and sodium borohydrate (NaBH4) was purchased from Panreac Química S.L.U. (Barcelona, Spain). DNA was used without further purification, since preliminary experiments showed that purification does not produce changes in experimental results. The absorbance ratio of DNA stock solutions at 260 nm and 280 nm was monitored and found to be between 1.8 and 1.9 (A260/A280 = 1.87), which indicates no protein contamination [40]. An agarose gel electrophoresis test using ethidium bromide indicated that the average number of base pairs per DNA molecule is above 10,000 bp. In order to have the biopolymer concentrations in base pairs, ds-DNA concentrations were determined spectrophotometrically at 260 nm from 13,200 M−1cm−1 DNA molar absorptivity [41]. The total concentrations of the DNA polynucleotide, the Tetracycline, 16-3-16 gemini surfactant, the gold nanoparticles functionalized with 16-3-16, Au@16-3-16, and the compacted nanosystem, Au@16-3-16/DNA-TC in a working solution will now be referred to as C_DNA_, C_TC_, C_16-3-16_, C_Au@16-3-16_ and C_Au@16-3-16/DNA-TC_, respectively. All solutions were prepared with de-ionized and autoclaved water (conductivity being less than 10^−6^ S·m^−1^), at a fixed ionic strength of 1.63 mM.

#### 2.1.1. Bacterial Lines and Culture Conditions

Commercial reference strains were used as controls. Specifically, Staphylococcus aureus ATCC^®^ 29,213 (Thermo Scientific, Lenexa, KS, USA) and Escherichia coli ATCC^®^ 25,922 (Thermo Scientific, Lenexa, KS, USA). Both bacteria were cultured under aerobic conditions at 37 °C. These strains are frequently used in antimicrobial resistance assays [42].

#### 2.1.2. Synthesis of 1,3-Propanediyl-bis-(dimethylhexadecylammonium bromide), 16-3-16

The gemini surfactant 16-3-16 used was synthesized and provided by the Institute of Heavy Organic Synthesis of Blachownia (Poland) and by Innovia Sp.z.o.o in Dobra (Poland) (see Supplementary Figure S1 in Supplementary Materials). Its CMC measured in situ is 0.022 × 10^−3^ M, which is in good agreement with previous measurements carried out by conductivity (CMC = 0.026 M [43], and CMC = 0.0255 M [44]). To prepare the surfactant solution necessary for the synthesis of the gold nanoparticle stabilized with 16-3-16, Au@16-3-16, a concentration of surfactant five times higher than its CMC in water (0.022 × 10^−3^ M) was employed. A sonicator was used to facilitate the dissolution of 16-3-16 in water solvent for 2 min. Once this was done, Milli-Q water was heated to 40 °C; then, maintaining this temperature, the solution was stirred continuously until it became crystalline, to ensure that the surfactant had completely dissolved. The surfactant must be at room temperature for use, so it was allowed to cool or was placed on ice to accelerate cooling.

#### 2.1.3. Synthesis of Au@16-3-16 Gold Nanoparticles

To prepare 16-3-16 functionalized gold nanoparticles, 300 μL of aqueous solution of HAuCl_4_ 23 mM, 99.9% purity was added to 30 mL of 16-3-16 surfactant 10^−4^ M and the mixture was stirred vigorously for 5 min in darkness, giving a yellow solution. Subsequently, 100 μL of a freshly prepared 0.4 M NaBH_4,_ 96% purity aqueous solution was added drop by drop to the previously prepared mixture and stirred moderately for 10 min in darkness, acquiring a reddish color. After that, the optimum condition to synthesize gold nanoparticles was found to be at 24 h of incubation time at 25.0 °C, followed by storage of the product at 5.0 °C. As a result, an aqueous solution of Au@16-3-16 nanoparticles at a 1.70 × 10^−7^ M concentration was obtained. In this study, we employed three formulations of Au@16-3-16 (Ni) for bacterial experiments prepared at different C_16-3-16_ concentrations of 51 nM, 74 nM and 130 nM, which were designated N_1_, N_2_ and N_3,_ respectively.

#### 2.1.4. Synthesis of Au@16-3-16/DNA-TC Nanocomplexes

To obtain Au@16-3-16/DNA-TC compacted nanocomplexes (C_i_), DNA/TC complexes were first prepared and the appropriate quantity of the synthesized precursor, Au@16-3-16, was employed to guarantee the maximum compaction state of the biopolymer in each case. Note that the range of relative nanoparticle-DNA concentrations for optimal biopolymer compaction was stablished between R = C_Au@16-3-16_/C_DNA_ = 0.74–1.3 × 10^−3^ (see AFM CD, DLS results in Section 3.1 and Section 3.2). Moreover, the DNA/TC complexes were prepared by mixing them for 2 min at room temperature, working under saturation conditions in order to transport the maximum amount of drug per nanocomplex (X = C_TC_/C_DNA_ = 0.5), using the intercalative DNA/TC complex as vehicle [45]. The prepared DNA/TC complex was gently stirred with Au@16-3-16 nanoparticles and incubated at 25 °C for 5 min. As a result, the position of the surface plasmon resonance (SPR) absorbance peak moved from 519 nm to 521–522 nm. This change was accompanied by an increase in the absorbance intensity of the nanoparticle after 24 h of stabilization and cold conditioning time, indicative of the formation of the nanocomplexes.

### 2.2. Methods

#### 2.2.1. UV/Vis Spectroscopy

Absorbance spectra were carried out using a CARY 500 SCAN UV−vis−NIR (Ultraviolet/Visible/Near Infrared) spectrophotometer (Varian, Markham, ON, Canada). Data were collected every 2 nm using a standard quartz cell with a path length of 10 mm. Wavelength accuracy and spectral bandwidth were ±0.3 nm and 0.5 nm, respectively. To study the stability of Au@16-3-16 and Au@16-3-16/DNA-TC nanosystems, changes in UV–vis spectra from 400 to 800 nm were followed over time and checked for at least 1 month.

#### 2.2.2. Circular Dichroism (CD) Spectroscopy

Electronic CD spectra were recorded with a BioLogic Mos-450 spectropolarimeter (Barcelona, Spain). A standard quartz cell with a 10 mm path length was used. The spectra were expressed in terms of molar ellipticity, [θ]. Scans were taken from 220 nm to 320 nm, working in the intrinsic CD region of DNA. For each spectrum, 5–10 runs were averaged at a constant temperature of 298.0 K with a 10 min equilibration before each scan. The interactions and conformational changes induced by the Au@16-3-16 nanoparticles in DNA/TC complexes were studied working at fixed C_DNA_ = 100 μM and C_TC_ = 50 μM concentrations and varying Au@16-3-16 concentrations, from 0.56 nM to 0.13 µM.

#### 2.2.3. Atomic Force Microscopy (AFM) Experiments

To obtain AFM micrographs in air, a Molecular Imaging Picoscan 2500 (Agilent Technologies, Las Rozas, Madrid, Spain) was employed. The resonance frequency and spring constant of silicon cantilevers (Model Pointprobe, Nanoworld Neuchâtel, Switzerland) was approximately 240 kHz and 42 N/m, respectively. The roughness of the sample was swept by the tip with a piezoelectric system in contact with the surfaces. All AFM images were recorded in tapping mode, with scan speeds of about 0.5 Hz and data collection at 256 × 256 pixels. The acquired AFM images were flattened to remove the background slope [46]. For sample visualization, a 1% (*v*/*v*) APTES solution with a 20 min incubation time was used to modify the mica surface. Subsequently, the surface was washed with ultrapure water and air-dried. A total of 30 μL of Au@16-3-16 or of Au@16-3-16/DNA-TC nanocomplexes (C_DNA_ = 0.3 μM and C_TC_ = 1.35 μM) at different C_Au@16-3-16_ from 0.221 to 0.389 nM and R ratios (R = 7.4 × 10^−4^–1.3 × 10^−3^) was dropped onto this modified surface and incubated for 30 min. Finally, the sample was washed in depth with ultra-pure water followed by air drying for AFM visualization.

#### 2.2.4. Dynamic Light Scattering (DLS) and Zeta Potential Measurements

The size and distribution of the synthesized Ni and Ci nanoformulations were characterized by means of the DLS technique using a Zetasizer Model ZS-90 (Malvern, Worcestershire, UK). Samples were illuminated with a laser characterized by a fixed detection arrangement of 90° to the center of the cell area, and the intensity fluctuation in the scattered light was then analyzed. At least 5 size measurements were taken for each sample, and the relative error for the hydrodynamic diameter calculated to be <5%. For Zeta potential measurements, a Zetasizer Nano ZS from Malvern Instrument Ltd. (Worcestershire, UK) was used. A laser Doppler velocimeter (LDV) was used to measure the velocity of the particles and the Zeta-potential (ζ) values were calculated from the electrophoretic mobility. A DTS1060 polycarbonate capillary cell was used, and the number of repetitions for each sample was at least six. To prepare samples, C_16-3-16_ concentrations were varied, while C_DNA_ = 100 μM and C_TC_ = 50 µM concentrations were fixed.

#### 2.2.5. Transmission Electron Microscopy (TEM)

To obtain TEM images of Au@16-3-16 gold nanoparticles, a TEM-TALOS F200S high resolution electron microscope was employed. The sample was deposited on a copper grid coated with a carbon film, then air dried for at least two hours at room temperature. The resulting images were analyzed using ImageJ 1.52a software. From these measurements, Au@16-3-16 was found to have a diameter of (2.5 ± 1.0) nm (see Supplementary Figure S2).

For the visualization of precursor and compacted nanosystems in cell samples, a Zeiss electron microscope was used. Approximately 500 cells were visualized using different treatments, including controls without any reagent; that is, free TC, nanoparticles Au@16-3-16 (N_3_) and Au@16-3-16/DNA-TC compact nanosystems (C_3_). A 1.6% glutaraldehyde solution was used to fix the different cell groups, followed by sample cleaning using a cacodylatetrihydrated solution (0.1 M and pH: 7.4) for 1 h at room temperature and/or 277.0 K overnight. Subsequently, the sample was placed in the Automatic Sample Processor using a protocol of 33 h and 25 min. This process was followed by sample storage post-fixation with a 1% osmium tetroxide solution. To contrast and stain the samples, a 2% uranyl acetate solution was used. This was followed by sample dehydration and gradual embedding in epoxy resin. Finally, they remained at 343.0 K for 7 h for polymerization of the resins. Next, we proceeded to first perform semi-fine cuts with a glass sheet in a standard range of 300 nm. To determine the study areas, prior to making the ultra-fine sections, semi-fine sections were made and stained with toluidine blue and visualized with an optical microscope. Ultra-fine cuts less than or equal to 70 nm were then made with a diamond disc. Visualization of samples was carried out with a Zeiss Libra microscope, using 300 mesh copper grids. For more details, see the protocol followed by the research group in previous works [47,48,49]. Tests were performed at different times (2, 6 and 12 h) to check the internalization of the precursors and the nanosystems with their respective controls. Different samples were studied with observation of between 500 to 700 bacteria per experiment.

#### 2.2.6. Energy Dispersive Spectroscopy (EDS) Measurements

For the study of elementary components, we prepared cells fixed, treated and cut with the ultramicrotome. Next, a microanalysis of an ultrathin section of the sample was carried out using the electronic scanner of the Zeiss EVO LS15 microscope. To do this, we used energy dispersive spectroscopy (EDS), with which we determined the presence of gold in the sample.

#### 2.2.7. Gold Nanosystems Susceptibility Tests against Gram + and Gram − Reference Strains

Once the most stable nanosystems were selected, the comparative antibacterial efficacy on bacterial cultures was evaluated. Each bacterial system selected was explored in the presence of TC = 50 µM, N_1_, N_2_ and N_3_ 16-3-16 gold nanoparticles (C_16-3-16_ concentrations of 51 nM, 74 nM and 130 nM, respectively), and C_1_, C_2_ and C_3_ nanocomplexes (where C_DNA_ = 100 μM and C_TC_ = 50 µM were fixed and C_16-3-16_ concentrations were varied, being of 51 nM, 74 nM and 130 nM for C_1_, C_2_ and C_3_, respectively). In this way, the minimum dose necessary to inhibit bacterial growth was evaluated in each case. To evaluate the effectiveness of the selected nanosystems as most suitable on bacterial growth, determination of the Minimum Inhibitory Concentration (MIC) was carried out using the two-fold micro-dilution standard assay according to the protocol described by the European Committee for Tests of Antimicrobial Susceptibility. The cutoff values used for the interpretation of MIC results were taken from EUCAST [50,51]. For the assays, 96-well U-bottom plates were used, one row being used to assess the effect of the three nanosystems, another three for each nanoparticle concentration (not attached to TC) and another 7 rows for tetracycline (three nanosystems and 4 controls).

All of these were dispensed in the amount of 200 µL in each of the first wells of each row of the plate, followed by double dilutions in 100 µL of Müller Hinton broth that had been previously dispensed in the rest of the columns (2–12). To this end, 100 µL was taken from each well starting with the first, and passed to the well in the second column, leaving the product diluted by half. From this, 100 µL was passed to the third until the last, from which 100 µL was discarded in order to always end up with a final volume of 100 µL. Finally, 100 µL of a bacterial suspension with an optical density of 0.08–0.1 (approximately 105 cfu) was added to all wells. Specifically, the OD was 0.091 for *E. coli* and 0.096 for *S. aureus.* The plates, once covered, were incubated for 24 h at a temperature of 37 °C. After this period, a macroscopic control of the plates was performed, checking the dilution at which a button of bacteria was observed in the bottom. The plates were then shaken on a shaker, and once all the buttons had disappeared, each well was read spectrophotometrically in an ELISA reader at a wavelength of 540 nm.

To evaluate the MBC (Minimum Bactericidal Concentration), sterile calibrated loops were used to transfer 10 µL from the wells of the microtiter plates to Manitol and MacCokey agar (for *S. aureus* and *E. coli*, respectively). Only the first wells with a bacterial button in the bottom and the previous dilution (without button) were cultured. Results were read after 24 h of incubation at 37 °C.

In addition, disk diffusion tests to confirm the susceptibility of reference strains to tetracycline (30 µg) were performed by culturing the strains in Müller Hinton agar for 24 h at 37 °C (see Supplementary Figure S3).

## 3. Results and Discussion

### 3.1. Conformational Changes in DNA/TC Complexes Induced by Au@16-3-16 Cationic Nanoparticles: Au@16-3-16/DNA-TC Complex Formation

**Figure 1:**
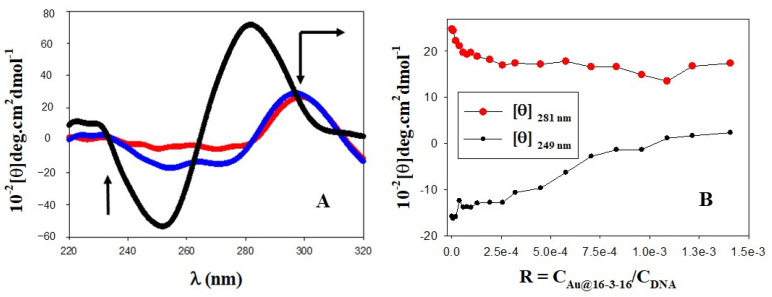
(**A**) CD spectra of DNA in the absence and in the presence of TC and gold nanoparticles. Arrows indicate the evolution of change in the molar ellipticity of the biopolymer. The spectra represented correspond to the following reagent concentrations: (i) C_DNA_ = 1.0 × 10^−4^ M, C_TC_ = 0 M and C_Au@16-3-16_ = 0 M (black spectrum); (ii) C_DNA_ = 1 × 10^−4^ M, C_TC_ = 5.0 × 10^−5^ M and C_Au@16-3-16_ = 0 M (blue spectrum); (iii) C_DNA_ = 1 × 10^−4^ M, C_TC_ = 5.0 × 10^−5^ M and C_Au@16-3-16_ = 1.3 × 10^−7^ M (red spectrum). (**B**) CD trend in molar ellipticity units ([θ]) of the system at fixed C_DNA_ = 100 µM and C_TC_ = 50 µM; C_Au@16-3-16_ variable.

Trojan horse-inspired delivery systems have been increasingly reported as effective strategies to efficiently combat and/or reinforce the efficacy of conventional antimicrobials against drug-resistant microbes, while also minimizing the side effects of treatment [52]. DNA is a genetic material that possesses high biocompatibility and low cytotoxicity, making it ideal for applications in biomedicine [53,54,55]. In this sense, the use of DNA biomolecules in the construction of new Trojan horse-inspired nanostructures for antimicrobial transport could confer great advantages in antimicrobial biodistribution, contributing to decreasing antimicrobial resistance. Furthermore, DNA nanostructures could be easily internalized within the cells and used effectively in a compact form for drug delivery purposes [56]. The binding of TC drugs with ds-DNA is mediated by electrostatic and hydrophobic forces, acting both as surface binder and intercalator [45]. As a result, perturbations in the secondary structure of DNA that could modify the biological and biochemical effects of TC are expected to occur. Moreover, the role of Au@16-3-16 nanoparticles in Au@16-3-16/DNA-TC complex formation and DNA conformational changes must be explored in order to verify suitable nanoparticle-polymer concentrations (R = C_Au@16-3-16_/C_DNA_) to ensure DNA compaction, and at the same time minimize the size of the transporter. To explore such conformational changes that bring about DNA-TC interactions, CD spectroscopy and AFM techniques can be used in combination. In this sense, the black spectrum in Figure 1 shows a CD spectrum of DNA in the right-handed B-form, where the intensity of both negative and positive peaks at 280 nm and 249 nm is similar. As we know, the intrinsic CD spectrum of DNA in the region of 220–320 nm can be modified by DNA interactions with ligands; these interactions are responsible for the stacking interactions between the DNA bases and changes in the helical superstructure of the polynucleotide [57]. When TC was added to the DNA system in the absence of gold nanoparticles (see Figure 1 in blue), a great decrease in the intensity of both bands was observed. These changes are coupled with a displacement of the positive band to a higher wavelength, indicating partial denaturation of the double stranded DNA and partial biopolymer compaction. Note that the behavior observed could be a consequence of the intercalation of TC into base-stacking, decreasing the right-handedness of the DNA [58,59,60]. Subsequently, addition of increasing C_Au@16-3-16_ concentrations to a fixed amount of DNA/TC complex progressively decreases the intensity of CD spectra (see Figure 1A in red and Figure 1B), bringing about full DNA compaction of the system and minimizing the size of the nanocomplexes in R range values of 17.4 × 10^−4^–1.3 × 10^−3^.

**Figure 2:**
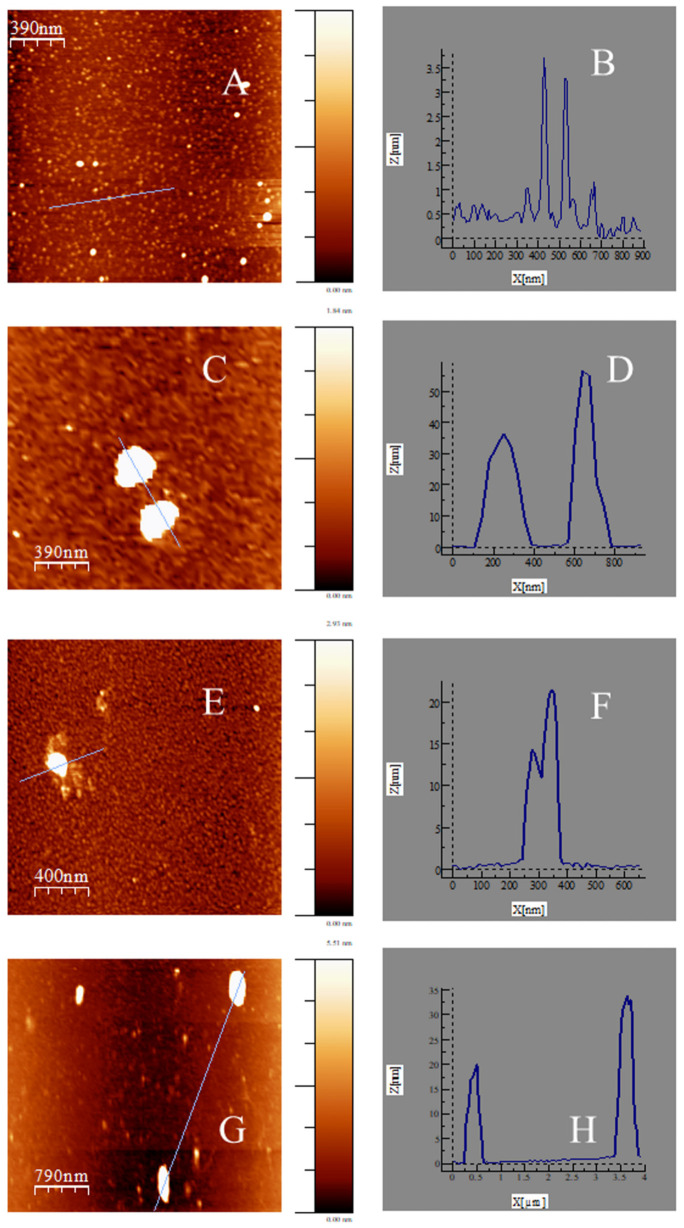
AFM topography images of Au@16-3-16 nanoparticles and Au@16-3-16/DNA-TC compacted nanocomplexes adsorbed on an APTES-modified mica surface in cacodylate buffer (I = 1.63 mM and pH = 7.4), under different reaction conditions (C_DNA_ = 0.3 μM, C_TC_ = 1.35 μM): (**A**) Au@16-3-16, C_Au@16-3-16_ = 17.0 nM, C_DNA_ = 0.0 μM, C_TC_ = 0.0 μM; (**C**) Au@16-3-16/DNA-TC, C_Au@16-3-16_ = 0.221 nM; (**E**) Au@16-3-16/DNA-TC, C_Au@16-3-16_ = 0.289 nM; (**G**) Au@16-3-16/DNA-TC, C_Au@16-3-16_ = 0.389 nM. Panels (**B**,**D**,**F**,**H**) correspond to the cross-sectional analysis of heights along the selected lines for images (**A**,**C**,**E**,**G**), respectively.

On the other hand, the structure and morphology of Au@16-3-16 nanoparticles and Au@16-3-16/DNA-TC compacted nanocomplexes was complementarily characterized via the ultrasensitive AFM technique (see Figure 2). Figure 2A,B show that the gold nanoparticles are spherical. The mean size of Au@16-3-16 nanoparticles is (2.7 ± 0.7) nm, taking into account analysis of the heights in z-direction. However, this value must be confirmed by TEM and DLS measurements. Note that the diameters of distinct nanosystems were analyzed in the z-direction to avoid the uncertainties observed in the x–y measurements due to image convolution, with the tip diameter obtained using the AFM technique [61,62].

As regards the morphology of Au@16-3-16/DNA-TC nanocomplexes, overall the shape was found to be roughly spherical and flattened, in some cases with some attached DNA/TC chains protruding outward (see Figure 2C,E). It is important to note that at C_Au@16-3-16_ = 0.389 nM (R = 1.298 × 10^−3^) in the C_3_ configuration, the compaction of the complex is fully accomplished, showing no traces of extended DNA strands along the structure, in accordance with CD results. Additionally, the sizes of all the complexes depend significantly on the R ratio in the z-direction, varying in the range of 55–20 nm, and showing a decrease in the size of the nanocomplexes and compaction tendency with the increase in the proportion of C_Au@16-3-16_/C_DNA_. However, the size of the complexes in the x-y direction undergoes an opposite behavior, varying from 50 to 450 nm on average when the C_Au@16-3-16_/C_DNA_ proportion is increased. This fact can be explained taking into account the possible formation of large flattened aggregates on the AFM plate. Apparently, the interaction of the hydrophilic and negatively charged APTES-functionalized mica surface with the hydrophilic and positively charged Au@16-3-16 nanoparticles could promote surface interaction among adjacent nanoparticles, contributing to the lateral expansion of the latter on the mica surface [49]. Thus, additional DLS studies must be carried out in order to more precisely measure the size of these nanostructures.

**Figure 3:**
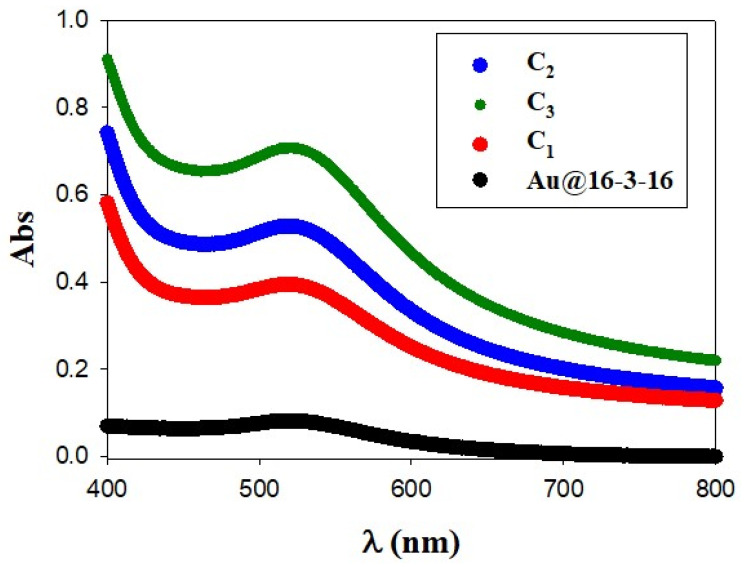
Absorbance spectra of Au@16-3-16 and Au@16-3-16/DNA-TC nanocomplexes under different reaction conditions in cacodylate buffer (I = 1.63 mM and pH = 7.4). Absorbance intensity increases from Au@16-3-16 precursor, C_Au@16-3-16_ = 17.0 nM (in black) to C_1_ (in red), C_2_ (in blue) and C_3_ (in green) compacted nanocomplexes. Spectra were measured after 24 h of stabilization.

Finally, to completely characterize complex formation, the UV–vis spectra of Au@16-3-16 and Au@16-3-16/DNA-TC nanocomplexes after 24 h of stabilization and cold conditioning time were analyzed. The UV–visible plot for free 16-3-16 nanoparticles is shown in Figure 3 (in black), exhibiting a well-defined surface plasmon resonance (SPR) band with a maximum at λ_SPR_ = 519 nm. According to the Wolfgang correlation, λ_SPR_ = 512 + 6.53 × exp. (0.0216 × d), where d is the diameter of the gold core, a mean size of 3.2 nm for the gold core is expected, which is in good agreement with AFM results.

A slight displacement of the SPR maximum as well as a progressive increase in the absorbance intensity with the R ratio was displayed when DNA/TC complexes were added to the Au@16-3-16 nanoparticles, indicating that the formation of the compacted nanocomplexes was properly accomplished. Note that the shift to a longer wavelength and the moderate broadening of the SPR peak with respect to the precursor spectra is compatible with the increase in size of the nanocomplexes with the R ratio observed using the AFM technique. Regarding the hyperchromic effect observed for the intensity of SPR bands after Au@16-3-16 binding to DNA/TC complex, this could be related to the more regular and better dispersed morphology of the synthesized nanocomplexes. This change in the structural configuration of the nanoparticle may result in a highly sensitive, more regular and better-dispersed derivative [63].

### 3.2. Charge, Size and Stability of Au@16-3-16 and Au@16-3-16/DNA-TC Nanosystems

**Figure 4:**
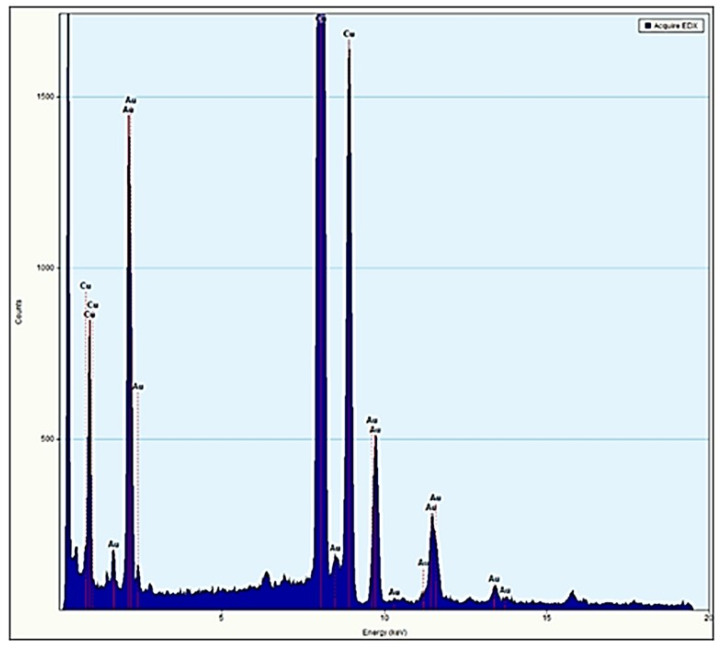
EDS-MET graph showing chemical composition of Au@16-3-16 gold nanoparticles. Only inorganic compounds were observed: Copper (Cu) and Gold (Au).

TEM measurements were carried out in order to more precisely identify the size of the gold nanoparticles (see Supplementary Figure S2). The evidence from these measurements indicates that Au@16-3-16 particles can be spherical in shape, having a diameter of (2.5 ± 1.0) nm. Furthermore, from EDS-MET measurements, it is possible to study the presence or absence of gold elements in the sample deposited on a copper microgrid (see Figure 4). Microanalysis confirms the presence of gold in the nanoparticles, which is detected together with the copper element from the grid. Note that other organic compounds present in the sample could not be visualized, since the microscope destroys organic compounds and only inorganic compounds can be observed.

**Figure 5:**
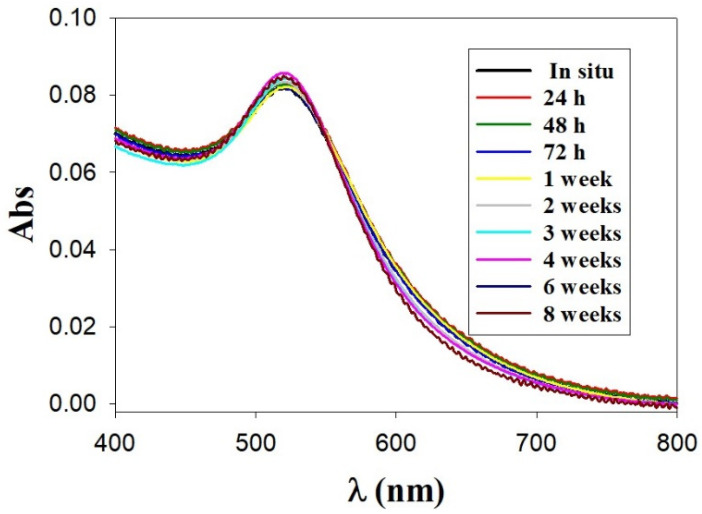
Stability study over time by UV-visible spectrophotometry for Au@16-3-16 nanoparticles.

On the other hand, one of the most important technological advantages of nanoparticles used as drug carriers is their high stability. This fact makes the possibility of variable routes of administration feasible, including oral administration and inhalation [64]. In this regard, the stability of distinct Au@16-3-16 and Au@16-3-16/DNA-TC formulations was explored by studying the possible modifications of the UV-vis spectra over time for at least one month. As an example of this, Figure 5 shows the UV-visible spectra of Au@16-3-16 nanoparticles, where the absorbance curves are practically superimposed, indicating their stability for up to two months.

Moreover, the same figure shows no evidence of broadening of the SPR peak, dismissing the possibility of significant aggregation processes in the nanoparticles [65]. Furthermore, Supplementary Table S1 in the Supplementary Materials shows similar results for the rest of the formulations studied after 24 h of stabilization, where the position of the maximum surface plasmon and the intensity of the absorbance signal were maintained over time for at least one month.

Analysis of the charge and size of nanosystems serves not only to characterize them, but also to measure their stability when dispersed in a specific solvent. In an applied electric field, charged species are attracted to the electrode of the opposite polarity, resulting in an electrostatic potential called zeta potential that represents the global charge at the nanoparticle surface. As is well known, a high positive or negative charge of around ±30 mV is considered optimum to attain physical colloidal stability [66]. As shown in Table 1, the zeta potential values of different systems studied are much higher than the abovementioned, guaranteeing their optimal stability. A well-defined zeta potential peak is observed for all samples studied, as can be seen from Supplementary Figure S4. The zeta potential of Au@16-3-16 nanoparticles is highly positive in connection with the charge of the gemini surfactant micelles that stabilize the nanosystem. In contrast to this, the charge of the DNA/TC complex is highly negative due to the negatively charged phosphate groups on the polymer backbone.

**Figure 6:**
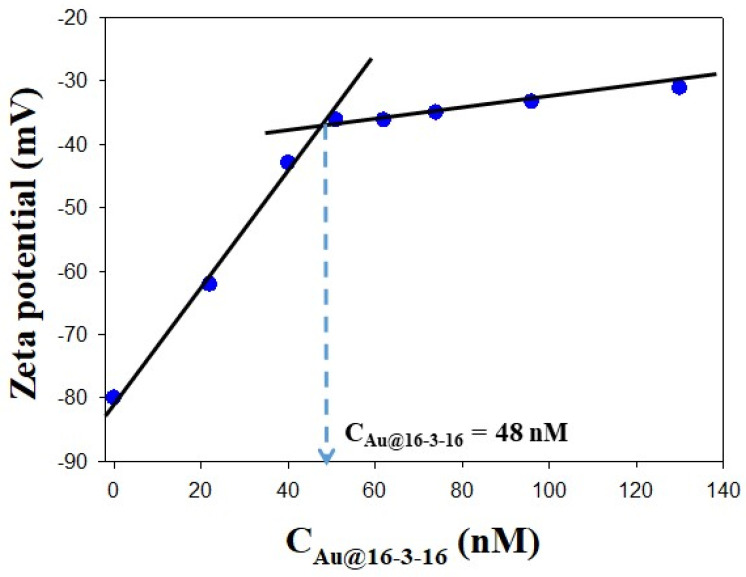
Plot of zeta potential values versus Au@16-3-16 concentration at fixed R = C_Au@16-3-16_/C_DNA_ = 1.298 × 10^−3^ and X = C_TC_/C_DNA_ = 4.5 ratios. Dots correspond to experimental zeta potential data. Arrow indicates an observed inflection point.

Figure 6 and Table 1 also show the zeta potential of Au@16-3-16/DNA-TC nanocomplexes as a function of the Au@16-3-16 nanoparticle concentrations. The zeta potential value of the complexes is determined by the charge ratio between the Au@16-3-16 nanoparticles and the DNA/TC complex. Initially, the zeta potential of the DNA/TC complex increases sharply from −80 mV to −36 mV (at C_Au@16-3-16_ = 48 nM), when an increasing quantity of positively charged gold nanoparticles is added. This behavior prior to the inflection point can be explained considering that the charges in the DNA/TC chains become shielded by the positively charged micelles that cover Au@16-3-16 nanoparticles [67,68]. However, as can be seen in the figure, further addition of C_Au@16-3-16_ after the inflection point increases the zeta potential more slowly. This change in the slope of the zeta potential plot vs. C_Au@16-3-16_ is due to a moderate conformational change that DNA/TC complexes undergo at high C_Au@16-3-16_. In fact, the variation of zeta potential depends not only on the charge ratio of the complexes, but also on biopolymer conformation. Thus, as the shear plane formed at the interface between the Sterns and diffuse layers of the double layer increases further away from the Au@16-3-16/DNA-TC complexes, the zeta potential decreases [69], in such a way that depending on the DNA conformation state, this shear plane will be located close to the nanoparticle surface for a compacted structure, and further away from the surface when the DNA is in a more extended conformation. Thus, the second decrease in zeta potential of nanocomplexes can be explained by taking into account that a more extended conformation of DNA/TC complex was adopted as C_Au@16-3-16_ concentration increased.

In order to clarify the nature of the different changes in DNA/TC conformation, we carried out dynamic light scattering (DLS) measurements on DNA/TC and Au@16-3-16/DNA-TC complexes. Since stability in terms of nanoparticle size is defined as the preservation of nanoparticle dimensionality during storage and/or an experiment [70], accurate DLS results reinforce previously discussed spectroscopic studies. The hydrodynamic diameters of the samples, taken from the position of the peaks of the size distribution by number function, are also collected in Table 1. The size of Au@16-3-16 nanoparticles is about 2.6 nm, showing a unimodal distribution (see Supplementary Figure S5A); this result is in good agreement with previously described TEM and AFM microscopy results. On the other hand, the size distribution of DNA/TC complexes shows that part of the biomolecule is already compacted by the tetracycline itself (see Supplementary Figure S5B). However, initially around 19% of the DNA was found to be in its extended form. Then, when Au@16-3-16 nanoparticles were added to the DNA/TC complex, two different regions were distinguishable in which a bimodal distribution appeared (see Table 1 and Supplementary Figure S5C–E). Moreover, the value of the hydrodynamic diameter shows that one of the populations has a smaller size than the other. Note that this result provides evidence of the existence of Au@16-3-16/DNA-TC nanoparticles in a compact form and correlates well with those obtained from CD and AFM measurement. Table 1 shows that in the first region, both populations show a marked decrease in size with increasing C_Au@16-3-16_ concentration. However, in the second region the opposite behavior is observed; that is, both populations increased slightly in size, supporting the zeta potential tendency. In addition, from C_Au@16-3-16_ = 51 nM, the percentage of the less abundant Au@16-3-16/DNA-TC population is almost negligible (<1%), with almost the entire nanocomplex being in its compact form. Taking into account that nanosystems with a reduced size (<100 nm) are able to cross bacterial membranes (>1 μm) successfully [25], the following study with bacteria was performed using different C_i_ nanoformulations (C_1_, C_2_ and C_3,_ see Table 1) prepared from C_Au@16-3-16_ = 51 nM, in order to ensure the maximum percentage of gold nanosystems in a more compacted state.

### 3.3. Results of Gold Nanosystems Susceptibility Tests against Gram + and Gram − Reference Strains

Previous studies have shown that gold nanoparticles possess strong antibacterial properties for Gram-negative and Gram-positive bacteria [71,72,73,74]. The most accepted explanation for the action of these particles is the production of reactive oxygen species (ROS) that arrest microbial growth by a process called the respiratory burst mechanism [74]. Other mechanisms of metal nanoparticles have been proposed, such as inactivation of enzymes through cysteine-binding, interaction of DNA with non-covalent binding, inhibition of electron transport within the cytoplasmic membrane, cell disruption via OH or other reactive species, or leakage of potassium from the cytoplasm into the extra cellular matrix [75].

Both reference strains were susceptible to tetracycline, with a diameter in the disk diffusion test of 27 mm for *S. aureus* (breakpoint according to EUCAST: 22 mm) and 23 mm for *E. coli* (breakpoint according EUCAST: 19 mm). Our results agree with other studies performed with the same reference strains [76,77,78].

**Figure 7:**
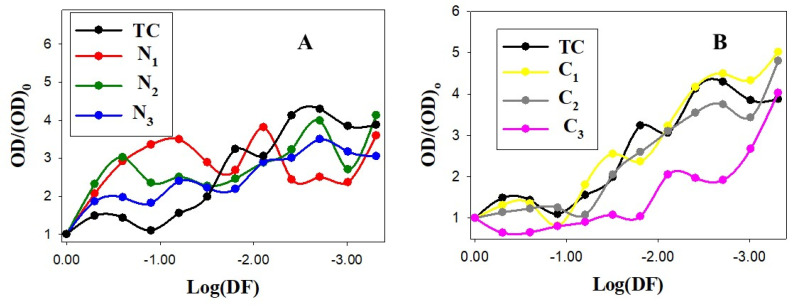
Spectrophotometry results for *S. aureus*. Dots correspond to experimental data and solid lines to trend lines. (**A**) TC control (C_TC_ = 50 µM) and Au@16-3-16 nanoparticles at different concentrations. N_1_, N_2_ and N_3_ formulations correspond to C_Au@16-3-16_ = 51 nM, 74 nM and 130 nM, respectively. (**B**) TC control (C_TC_ = 50 µM) and C_1_, C_2_, and C_3_ Au@16-3-16/DNA-TC nanosystems. (OD)_0_ corresponds to the optical density of the systems in the absence of dilution (see Section 2.2.7 for more details).

**Figure 8:**
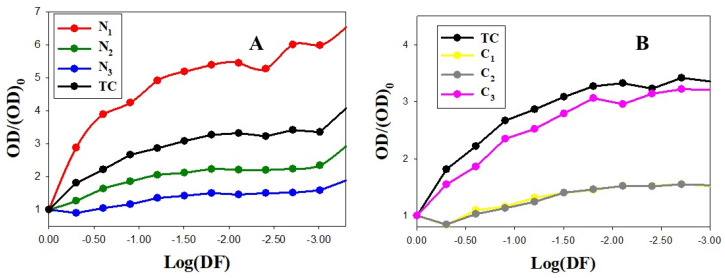
Spectrophotometry results for *E. coli*. Dots correspond to experimental data and solid lines to trend lines. (**A**) TC control (C_TC_ = 50 µM) and Au@16-3-16 nanoparticles at different concentrations. N_1_, N_2_ and N_3_ formulations correspond to C_Au@16-3-16_ = 51 nM, 74 nM and 130 nM, respectively. (**B**) TC control (C_TC_ = 50 µM) and C_1_, C_2_, and C_3_ Au@16-3-16/DNA-TC nanosystems. (OD)_0_ corresponds to the optical density of the systems in the absence of dilution (see Section 2.2.7 for more details).

Quantitative results measured by spectrophotometry for *S. aureus* and *E. coli* can be observed in Figure 7 and Figure 8, respectively. The X axis represents the dilution factor on the logarithmic scale, and the Y axis represents the relative optical density value measured at 540 nm.

For *S. aureus*, in N_1_, N_2_ and N_3_, a general increase in optical density from the first dilution can be observed (see Figure 7A). For C_1_, C_2_ and C_3_ nanosystems, the optical density is around 0.1 until the third dilution. However, we observed that the dilution in which the optical density increases from 0.1 in C_i_ nanoparticles depends on the nanosystem configuration: 0.0625 for C_1_, 0.03125 for C_2_ and 0.007125 for C_3_, while the increase for the TC control starts from 0.01525 (see Supplementary Figure S6). In Figure 7B, it can be observed that the values of the relative optical density are always below those of the TC control in all dilution factors explored for the C_3_ nanosystem. Importantly, this result demonstrates the efficacy of the C_3_ nanosystem for *S. aureus* inhibition, avoiding possible side effects. In addition, C_2_ exhibits slightly greater efficacy than the TC control, while C_1_ does not show appreciable differences. Note that the same TC concentration was used in the TC control and in the preparation of the C_1_, C_2_ and C_3_ nanosystems.

In the case of the Au@16-3-16 precursors, only the more concentrated nanoparticle (N_3_) showed the best effectivity with respect to the TC control at the highest dilution factor, but the optical density was higher when this nanosystem was not diluted (see Figure 7A). It can be assumed that this is the effect of the nanoparticle, as the button observed in the undiluted N_3_ well had a marked dark red color, as previously mentioned. However, at this point it is noteworthy that N_1_, N_2_ and N_3_ nanoparticles are free of TC. Hence, their antibacterial properties may be due to both the nature of the gold core and the hydrophobic and positively charged 16-3-16 gemini surfactant chains that surround the nanoparticles (see Table 1). In fact, Xiaoing Li et al., reported that 2 nm core cationic monolayer-protected AuNPs can interact with the cell membranes of Gram-positive and Gram-negative bacteria, resulting in the formation of distinct aggregation patterns and lysis of bacterial cells [71]. Moreover, Katarzyna et al. demonstrated that gold-functionalized magnetic nanoparticles, Fe_3_O_4_@Au, restricted the growth of pseudomonas aeruginosa bacteria, showing the action of the gold core as an antimicrobial agent [72]. In this sense, our results for Au@16-3-16 gold nanoparticles in *S. aureus* are in line with those previously described in the bibliography, showing remarkable antibacterial properties.

In Figure 8, we observe that optical density increases less markedly when the degree of dilution for Ci, N_2_ and N_3_ nanosystems increases than in the case of the TC control, preventing *E. coli* growth to a greater extent. However, the less concentrated N_1_ nanoparticles showed a decreased effect from the first dilution. In spite of the higher resistance of *E. coli* to TC in comparison with the *S. aureus*, the results in Figure 7 and Figure 8 show that, except for TC control and N_1_ nanoparticles, the relative optical density reaches higher values at the final dilution factor for *S. aureus* than for *E. coli* ((OD/(OD)_0_ in *E. coli*: 2.95, 1.90, 1.82, 1.70 and 3.30 for N_2,_ N_3_, C_1_, C_2_ and C_3_, respectively, vs. (OD/(OD)_0_ in *S. aureus*: 4.13, 3.05, 5.01, 4.80 and 4.02).

In the case of Ni nanoparticles, this fact can be explained by taking into account the physicochemical surface characteristics of both bacteria that play a key role in bacterial adhesion and aggregation. That is, the zeta potential of *E. coli* at physiological pH is more negative than that of *S. aureus* bacteria under the same conditions [79]. Thus, considering the high positive charge of Ni nanoparticles (see Table 1), we expect a higher electrostatic affinity of N_i_ particles for *E. coli*, improving their bactericidal effectiveness. In the case of C_i_ nanosystems as a TC transporter, the behavior is more complex, but we can consider the biomimetic environment conferred by the DNA biopolymer that can act as a Trojan horse, improving antimicrobial biodistribution. Furthermore, gold particles have also been demonstrated to enhance the antibacterial properties of antibiotics or to act synergistically [80]. Hence, the antibacterial properties of TC are usually enhanced in Ci nanosystems, and this effect is more evident in bacterial systems such as *E. coli* that present greater resistance to the use of tetracyclines as an antibacterial agent.

On the other hand, MIC results for *S. aureus* and *E. coli* (see Supplementary Figures S6 and S7) showed no bacterial button for *S. aureus* in the first column (no dilution). Buttons began to appear from the second column at 50% dilution in most of the rows, except for the more concentrated nanoparticles (N_3_), where the button was not perceptible until the fourth column (1/8 dilution). However, *E. coli* showed buttons from the second column on. In both plates, dark red buttons were observed in undiluted nanoparticle and more concentrated nanosystems (C_3_ and N_3_). These buttons showed stronger inhibition of the 16-3-16 nanoparticles and nanosystems, while the white buttons perceptible in the rest of the wells were due to bacterial precipitation. The MIC values of distinct nanosystems and TC control are collected in Table 2. The initial concentration (first column of each row) of TC in this study was 5.05 mg/L. This is applicable for the TC control and for the three C_i_ nanosystems. We find inhibition in the first column (5.05 mg/L), but not in the second (see Supplementary Figure S7), in accord with results described by Sodagari et al. [81], who reported in a study with *E. coli* that only 51% of the strains were sensitive to the doses initially used in this study (IC_50_ = 8). Other studies with *E. coli* [82] reported a IC_50_ value of 32 for TC. According to EUCAST [50,51], the IC_50_ value for TC in *E. coli* is 8 mg/L.

Regarding *S. aureus*, the IC_50_ value for TC also varies among studies. The EUCAST IC_50_ value for TC in *S. aureus* is 0.5 mg/L, but this was increased to 1 mg/L in a study performed by McDougall et al. [83] with cows in New Zealand, reaching 32 mg/L in another study with milk samples in Brazil [84]. In our study, the minimum inhibitory concentration (MIC) value for TC was 5.05 mg/L in TC control and C_i_ nanosystems. In the case of Ni nanoparticles, as they are free of TC, the MIC values can be calculated considering that Au@16-3-16 nanoparticles can properly act as an antibiotic. Thus, the MIC values are in the range of nanomolar concentration, revealing its great effectiveness. Moreover, this effect is most notable in the case of N_3_ in *S. aureus*, where this value was reduced to 16.2 nM, showing the greater efficiency of this nanoparticle.

Note that in order to check the bactericidal properties of the nanosystems and to compare their effectiveness in avoiding antibiotic resistance, we fixed the quantity of antibiotic in all the experiments. Moreover, DNA/TC complexes were prepared working under saturation conditions in order to transport the maximum amount of drug per nanocomplex, taking into account the value of the equilibrium binding constant of the intercalative complex (K_a_ = 1.2 × 10^7^ M^−1^) [45]. Thus, antimicrobial activity was measured using minimum inhibitory concentration as the key parameter. According to the results shown in Table 2, the effectiveness of both the nanoparticles (Ni, synthesized without TC), and the nanocomplexes (Ci, formed by the prepared DNA/TC complex and gold nanoparticles) seems evident. Upon comparison with other published studies, our results show a similar or lower MIC. For instance, the MICs of biosynthesized AgNPs for *E. coli* and *S. aureus* were 6.25 and 50 mg/mL, respectively [85]. Another study showed antimicrobial agents such as biosynthesized ZnONPs, where the MICs were 8 and 4 mg/mL against *E. coli* and *S. aureus*, respectively [86]. Another antimicrobial agent was phenyllactic acid, where the MIC was 2.5 against *E. coli* [87]. Another study showed for green synthesized AgNPs a MIC of 6.25 μg/mL in *E. coli* [88]. Note that these studies were carried out with seeding on mannitol agar plates. Thus, from those wells where growth inhibition was observed (immediately before those where there was a bacterial button) mannitol agar plates were seeded and the number of resulting colonies was checked after a new incubation of 24 h at 37 °C.

Taking into account the suitable physicochemical parameters of N_3_ and C_3_, and the in vitro viability of these nanoformulations in both *Staphylococcus aureus* and *Escherichia coli* bacteria, we take N_3_ and C_3_ as model systems to study their internalization features in both types of bacteria.

With reference to the Minimum Bactericidal Concentration (MBC), bacterial growth was observed in all wells except those in the first column (undiluted nanosystems or controls), except for Control nanoparticle N_2_ in the *E. coli* test. Supplementary Figures S8 and S9 show the bacterial growth in first (not diluted) and second column (first dilution) for *S. aureus* and *E. coli*, respectively. The absence of this bactericidal effect from the first dilution may be due to the fact that Tetracycline is a bacteriostatic antibiotic, not bactericidal [16].

### 3.4. Internalization of Au@16-3-16 and Au@16-3-16/DNA-TC Nanosystems

To verify the interaction and internalization of the precursors as well as the nanosystems, treatments were carried out at 6, 12 and 18 h. To do this, the cultured elements were collected and subjected to centrifugation to collect the pellet. In accordance with the results obtained in the MIC and spectrophotometry studies, treatments were carried out using both the precursor and the most concentrated nanosystems, N_3_ and C_3_, respectively. In all cases, controls without treatment and treatment with only TC were carried out. Photomicrographs were taken with low contrast to highlight the gold nuclei that make up the nanoparticles, against dense organelles such as ribosomes.

**Figure 9:**
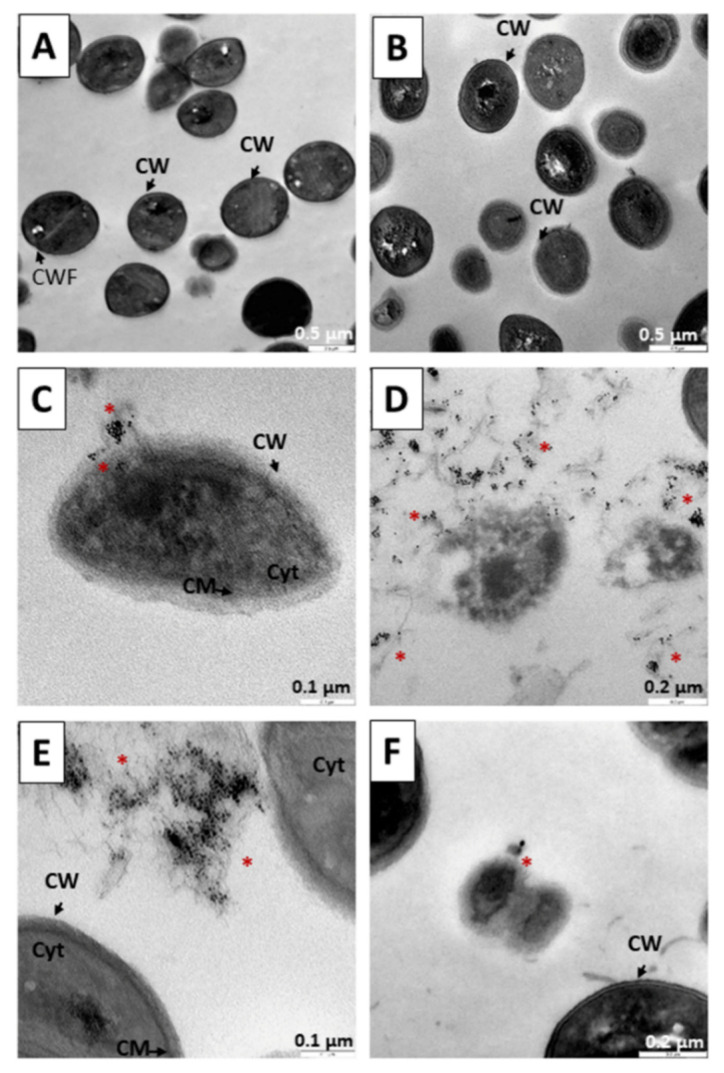
TEM microphotograph of *Staphylococcus aureus* after 6 h of treatment. (**A**) Control bacteria without treatment. (**B**) Bacteria treated with TC. (**C**,**D**) Treated with the N_3_ precursor. (**E**,**F**) Bacteria treated with the C_3_ nanosystem. Asterisks (*) indicate the presence of metallic nuclei compatible with the gold cores of the nanoparticles. Abbreviations: CW: Cell wall CWF: Cell wall in formation. CM: Cytoplasmic membrane. Cyt: Cytoplasm.

Figure 9 shows the results in a culture of *S. aureus* after 6 h. Figure 9A shows the absence of elements compatible with nanoparticles; the morphology of the bacteria is normal and bacterial divisions with evidence of the formation of a new cell wall can be observed. In Figure 9B, only TC treatment was performed, and no drastic reduction of cells was observed at 6 h. In Figure 9C,D, the precursor N_3_ was administered for 6 h. A large number of rounded structures compatible with the gold core of the nanoparticles are observed. Figure 9E,F, the bacteria affected by the 6-h treatments with the C_3_ nanosystem are shown. As mentioned above, gold nanoparticles possess strong antibacterial properties for both Gram-negative and Gram-positive bacteria [71,72,73,74]. As these Gram-positive bacteria, possibly due to the positively charged N_3_ producing structural damage, allow the internalization of nanoparticles and cause an induction of oxidative stress by the formation of reactive oxygen species, they release metal ions, also causing protein and enzyme dysfunction as well as signal transduction inhibition, including genotoxicity [89,90,91,92,93,94,95,96]. C_3_ is a nanosystem with a negative charge; therefore, an electrostatic interaction may occur, causing internalization by endocytosis. Once inside, the reversion of the elements of the nanosystems into their components produces a toxicity mechanism similar to that described with N_3_, releasing reactive oxygen species and metal ions, as well as causing dysfunction of proteins and enzymes, along with inhibition of signal transduction, including genotoxicity [89,90,91,92,93,94,95,96].

**Figure 10:**
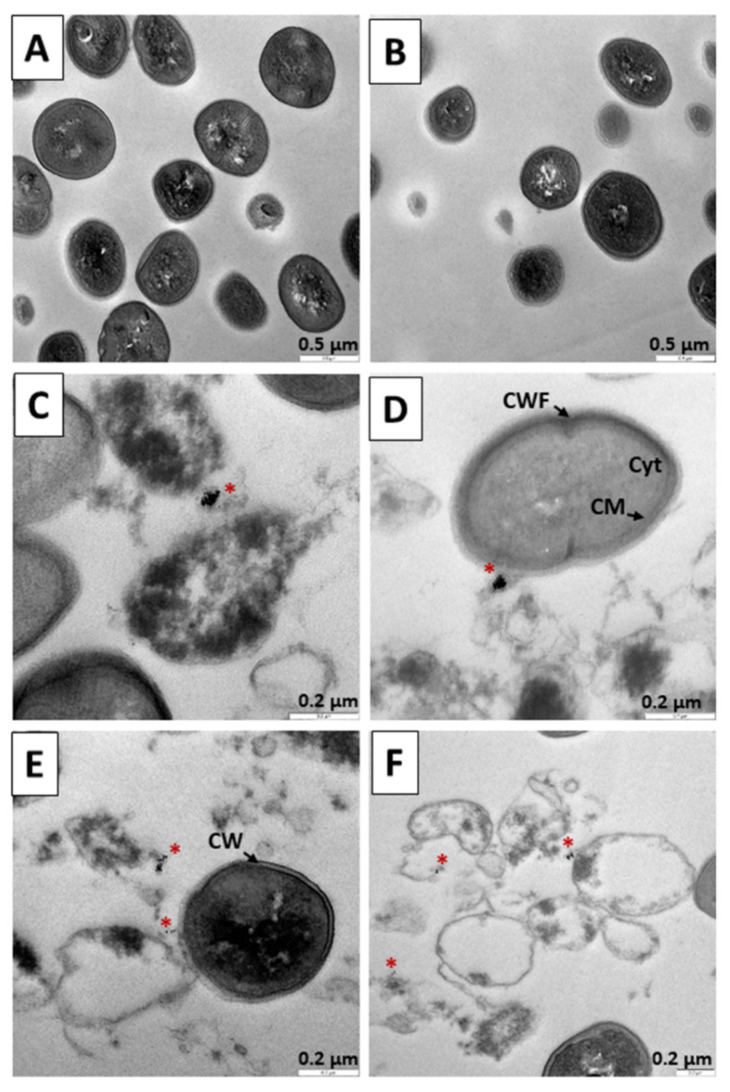
TEM microphotograph of *Staphylococcus aureus* after 12 h of treatment. (**A**) Control bacteria without treatment. (**B**) Bacteria treated with TC. (**C**,**D**) Treated with the N_3_ precursor. (**E**,**F**). Bacteria treated with the C_3_ nanosystem. Asterisks (*) indicate the presence of metallic nuclei compatible with the gold cores of the nanoparticles. Abbreviations: CW: Cell wall. CM: Cytoplasmic membrane. Cyt: Cytoplasm.

Figure 10 shows treatments performed on *S. aureus* for 12 h. Cells without treatment as a control are shown in Figure 10A. In Figure 10B, bacteria were subjected to TC treatment for 12 h, and reduction of bacteria was observed. In Figure 10C,D, precursor N_3_ was administered, and again interaction with N_3_ (Figure 10C,D) and the involvement of bacterial bodies was observed (Figure 10C). The greatest evidence of the destructive action of the nanosystems is observed in Figure 10E,F, where after 12 h of treatment with the C_3_ nanosystem, we observed significant destruction of the cell bodies (Figure 10E,F). The mechanisms used by both N_3_ and C_3_ are similar to those described in the 6-h experiments, the bacterial destruction being more evident here (Figure 10C–F).

**Figure 11:**
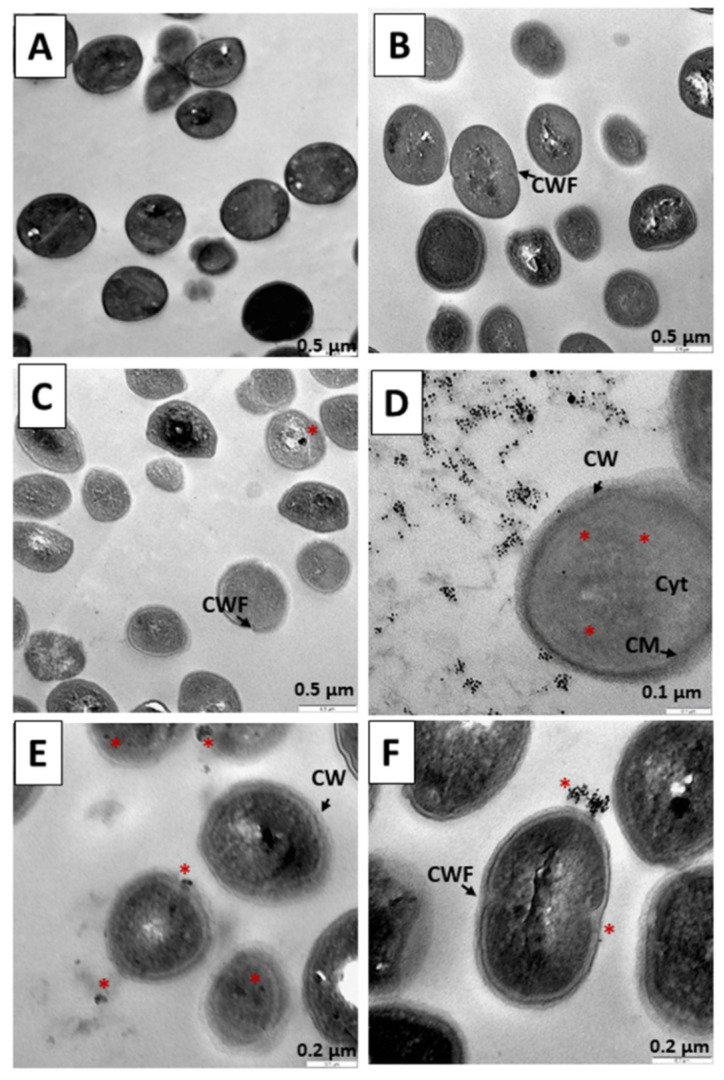
TEM microphotograph of *Staphylococcus aureus* after 18 h of treatment. (**A**) Control bacteria without treatment. (**B**) Bacteria treated with TC. (**C**,**D**) Treated with the N_3_ precursor. (**E**,**F**) Bacteria treated with the C_3_ nanosystem. Asterisks (*) indicate the presence of metallic nuclei compatible with the gold cores of the nanoparticles. Abbreviations: CW: Cell wall CWF: Cell wall in formation. CM: Cytoplasmic membrane. Cyt: Cytoplasm.

Figure 11 shows treatments performed on *S. aureus* for 18 h. Cells without treatment used as a control are shown in Figure 11A. In Figure 11B, the bacteria were subjected to TC treatment for 18 h, with structural damage observed in some of the bacteria. In Figure 11C,D, the precursor N_3_ was administered, and again great interaction with N_3_ (Figure 11C) and the great attraction of N_3_ around the bacterial bodies was observed (Figure 11D). In Figure 11E,F, the performance of the C_3_ nanosystem can be observed after 18 h. In the preparation, there is destruction of bacterial bodies (Figure 11E) and accumulation of rounded bodies compatible with the gold cores of the nanoparticles that make up the nanosystem. The mechanisms used by both N_3_ and C_3_ are similar to those described in the 6 and 12 h experiments, bacterial destruction being more evident here (Figure 11D,F).

**Figure 12:**
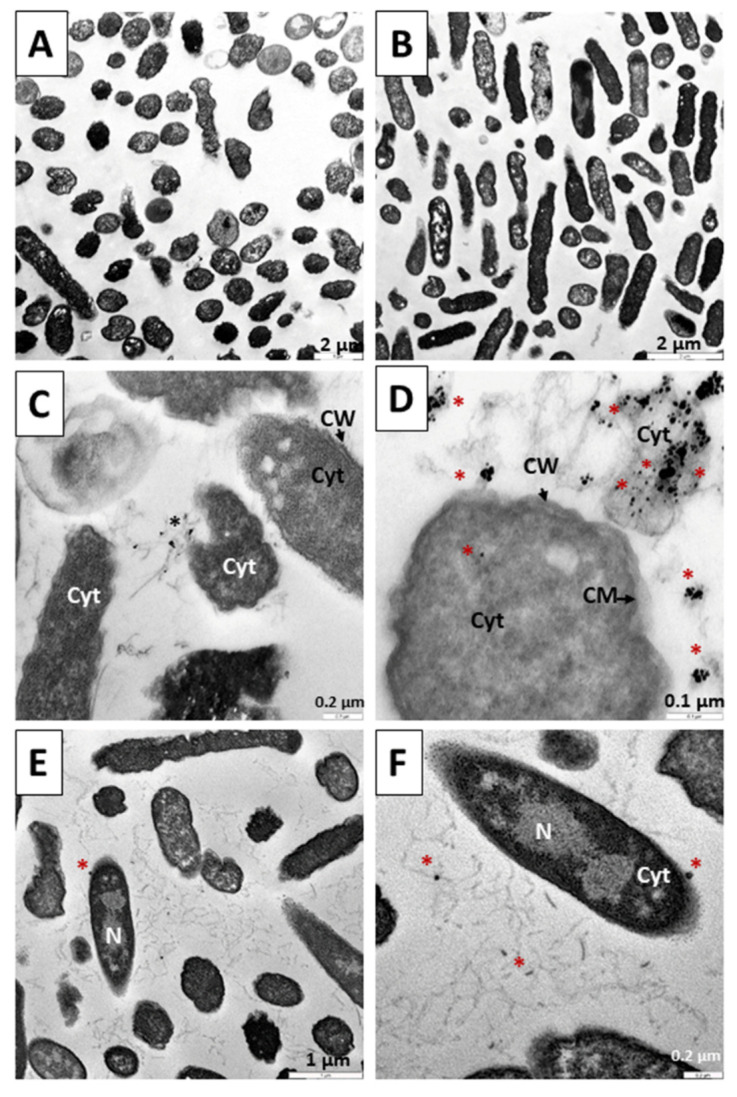
TEM microphotograph of *Escherichia coli* after 6 h of treatment. (**A**) Control bacteria without treatment. (**B**) Bacteria treated with TC. (**C**,**D**). Treated with the precursor of the N_3_ nanosystem. (**E**,**F**). Bacteria treated with the C_3_ nanosystem. Asterisks (*) indicate the presence of metallic nuclei compatible with the gold cores of the nanoparticles. Abbreviations: CW: Cell wall CM: Cytoplasmic membrane. Cyt: Cytoplasm.

Figure 12 shows the electron microscope microphotographs of a culture of *E. coli*, a Gram-negative bacterium. Figure 12A shows a control population without treatment at 6 h, where a normal morphology of these bacilli is observed. In Figure 12B, we can see the result of applying TC treatment for 6 h; a drastic reduction in the number of bacteria is not observed. Figure 12C,D show the result of the application of the N_3_ nanoparticle after 6 h. In this case, an electrostatic interaction between the bacterial wall and the nanoparticle possibly takes place, producing its internalization by endocytosis. This would cause damage to membrane proteins, induction of oxidative stress due to the formation of reactive oxygen species, release of metal ions, dysfunction of proteins and enzymes, and inhibition of signal transduction, although genotoxicity-type damage can also occur [89,90,91,92,93,94,95,96]. In Figure 12D, the damage caused by the contact of the precursor with the bacterium wall can be observed, causing a destruction of the cell cover and of the interior (Figure 12E). Figure 12F shows the effect of treatment with the C_3_ nanosystem for 6 h, showing cell debris with the release of small dense nuclei compatible with the gold nuclei of the nanoparticles that form the nanosystems.

**Figure 13:**
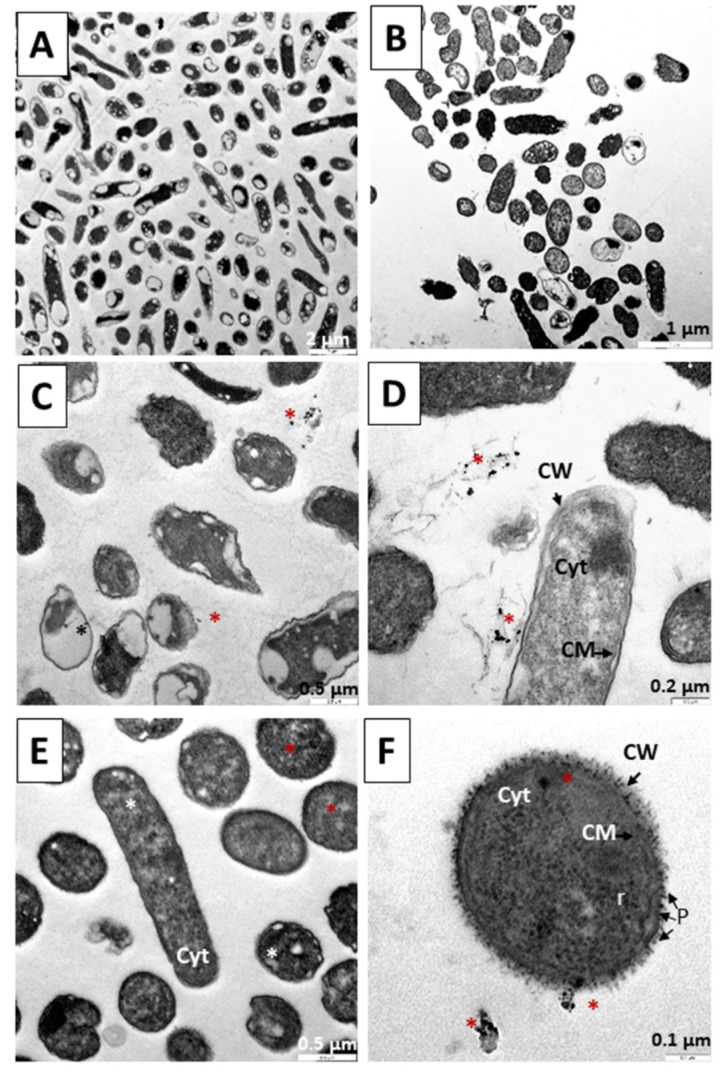
TEM microphotograph *Escherichia coli* after 12 h of treatment. (**A**) Control bacteria without treatment. (**B**) Bacteria treated with TC. (**C**,**D**). Treated with the precursor of the N_3_ nanosystem. (**E**,**F**). Bacteria treated with the C_3_ nanosystem. Asterisks (*) indicate the presence of metallic nuclei compatible with the gold cores of the nanoparticles. Abbreviations: CW: Cell wall CM: Cytoplasmic membrane. Cyt: Cytoplasm.

Figure 13 shows the microphotographs of an *E. coli* culture after 12 h of treatment. In Figure 13A, a control population without treatment is shown at 12 h, where a normal morphology of the bacterial bodies is observed. In Figure 13B, we can see the result of applying a TC treatment for 12 h, with a drastic reduction in the number of bacteria. In Figure 13C,D, we can observe the result of the application of the N_3_ nanoparticle after 12 h, with affected bacterial bodies and remains of the destructive effect caused by N_3_. In Figure 13E,F, the cell bodies attacked by the C_3_ nanosystem are shown. The mechanisms used by both N_3_ and C_3_ are similar to those described in the 6 h experiments, the bacterial destruction being more evident here.

**Figure 14:**
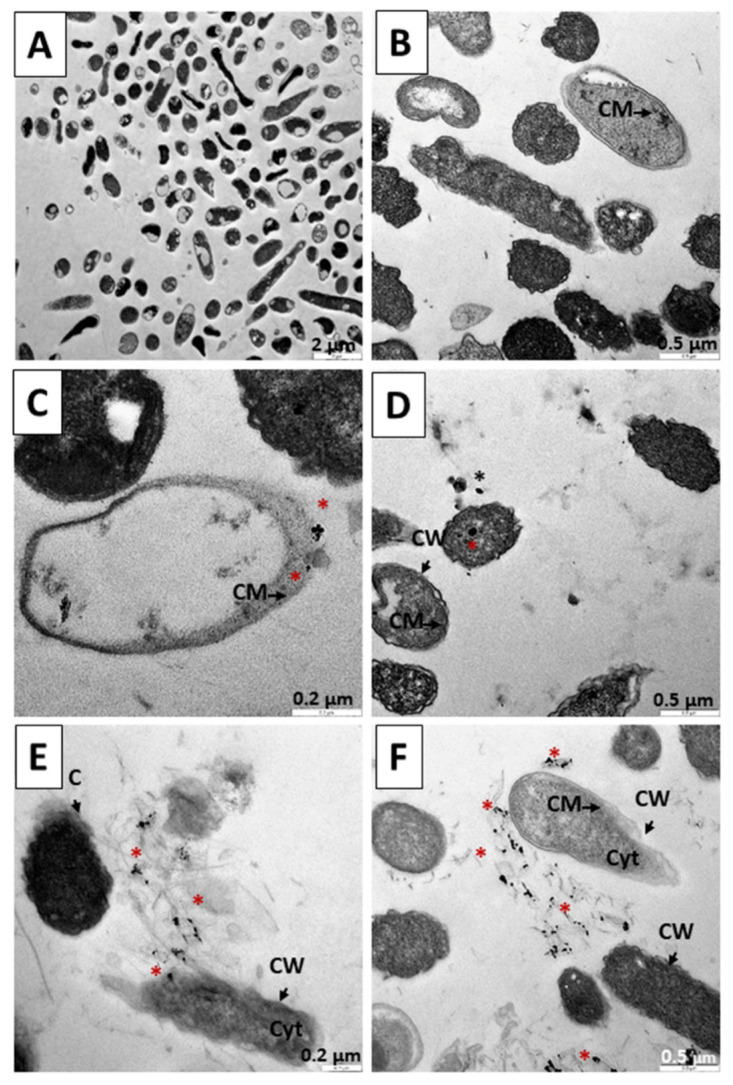
TEM microphotograph of *Escherichia coli* after 18 h of treatment. (**A**) Control bacteria without treatment. (**B**) Bacteria treated with TC. (**C**,**D**). Treated with the precursor of the N_3_ nanosystem. (**E**,**F**). Bacteria treated with the C_3_ nanosystem. Asterisks (*) indicate the presence of metallic nuclei compatible with the gold cores of the nanoparticles. Abbreviations: CW: Cell wall CM: Cytoplasmic membrane. Cyt: Cytoplasm.

Finally, Figure 14 shows the results of the application of treatments for 18 h. In Figure 14A, a control population without treatment is shown at 18 h, where a normal morphology of the bacterial bodies is observed. In Figure 14B, we can see the result of applying TC treatment for 18 h, with a great reduction in the number of bacteria. Similar to the 12 h treatment, in Figure 14C,D we can observe the result of the application of the N_3_ precursor of the C_3_ nanosystem after 18 h, with the affected bacterial bodies and remains caused by the destructive effect of N_3_. Likewise, in Figure 14E,F we can verify the devastating effects of C_3_ treatment for 18 h on the bacteria: bacterial remains can be observed throughout the preparation, with the release of dense particles compatible with the gold cores that form the nanoparticles. The mechanisms used by both N_3_ and C_3_ are similar to those described in the 6 and 12 h experiments, bacterial destruction being more evident here (Figure 14C–F).

Differentiation between Gram + and Gram − bacteria is based on the different characteristics of the cellular walls between both groups of bacteria; their names derive from the staining technique developed by the bacteriologist Hans Christian Gram [97]. In terms of structural properties, the main difference lies in peptidoglycan, better known as murein, one of the main constituents of the cellular wall, which forms a thick layer in Gram +, but a thin layer in Gram −. Thus, the Gram-positive bacteria have a thick peptidoglycan layer and no outer lipid membrane, whereas the Gram-negative bacteria have a thin peptidoglycan layer and an outer lipid membrane [98]. On the other hand, Gram − bacteria have a higher negative charge than Gram + bacteria [90]. The lipopolysaccharide in the outer layer of the lipid bilayer has more charge per unit surface than other phospholipids in Gram − bacteria, thus making them highly negative in charge [91,98]. Taking into account the charge properties of the bacteria, the great interaction between them and highly charged cationic N_3_ gold nanoparticles is justified, in such a way that an electrostatic interaction between the bacterial wall and the nanoparticles facilitates the internalization process of N_3_ or C_3_ in the case of the organism *E. coli* or *S. aureus*, respectively. However, when the electrostatic charge is similar in nature for both the bacterial wall and the nanoparticle (C_3_ or N_3_ in the case of *E. coli* or *S. aureus*, respectively), internalization is promoted by endocytosis. Through any of these mechanisms, once inside the bacteria, there is an induction of oxidative stress due to the formation of reactive oxygen species and release of metal ions, also causing the dysfunction of proteins and enzymes as well as inhibition of signal transduction, including genotoxicity [89,90,91,92,93,94,95,96]. This damage becomes more evident after 18 h of incubation.

## 4. Conclusions

Gold nanoparticles possess exceptionally useful properties, such as high stability, low toxicity and good biocompatibility, making them ideal for drug administration and use in different types of patients with various pathologies. It is extremely important to administer an adequate dose of antibiotic; in this way, bacterial resistance to antibiotics is avoided and the possible damage that high doses of medicine can cause in the human body is prevented. The gold nanoparticles used in this study are highly stable, and their synthesis is a simple process. Moreover, the use of the gemini surfactant as a stabilizing agent confers high stability to our system, and the high positive charge permits favorable electrostatic interaction with the DNA/tetracycline complex and the ability to induce DNA compaction. The antibiotic tetracycline is a good candidate for this purpose; it acts against a large number of bacterial infections, and it is unusual for bacteria to develop resistance to the antibiotic. Moreover, it is fat–soluble and has the ability to reach any body tissue. However, the dose of the antibiotic must be controlled due to its toxicity at high concentrations. Stability studies using ultraviolet–visible spectrophotometry show that the 16-3-16 based gold nanosystems N_i_ and C_i_ are stable over time. TEM microscopy studies allowed us to identify gold in the sample composition. In addition, TEM and AFM microscopies also allowed us to determine the average size of our nanoparticle and observe its morphology. Finally, our results show that the nanosystem composed of the Au@16-3-16 nanoparticle and the DNA/TC complex is appropriate for medical use. Moreover, Au@16-3-16 nanoparticles act as antibiotics by themselves in the absence of TC, given the fact that gold nanoparticles possess strong antibacterial properties for both Gram-negative and Gram-positive bacteria, as well as the high positive charge of the Ni nanoparticles and hydrophobic character conferred by the 16-3-16 surfactant hydrophobic chains. It is expected that it can be applied to patients with various types of pathologies with numerous benefits, including the reduction of derived side effects. The nanosystems showed a higher effectivity in inhibiting Gram-positive and Gram-negative bacteria compared with TC. Differences in the interaction of the precursors and the generated nanosystems were observed by TEM microscopy, as demonstrated in Gram-positive and Gram-negative bacteria, possibly due to membrane damage or electrostatic interaction with internalization by endocytosis. In internalization experiments, depending on the treatment application time, greater bacterial destruction was observed for both the N_i_ precursors and the C_i_ nanosystems tested after 18 h of incubation time. Gold-TC nanosystems may be an answer to the necessity for reduction of antibiotics, allowing a decrease of the amount used, especially in Gram-positive bacteria.

## Figures and Tables

**Table 1:** Analysis of zeta potential and size of Au@16-3-16 nanoparticles, DNA/TC and Au@16-3-16/DNA-TC complexes in cacodylate buffer (I = 1.63 mM and pH = 7.4), working at fixed C_DNA_ = 100 μM and C_TC_ = 50 μM concentrations.

	Sample	Sample Composition	Zeta Potential (mV)	Size (nm); Population %
	1	Au@16-3-16	(51.3 ± 1.9)	(2.6 ± 0.3)
**Region 1**	2	DNA/TC	(−80 ± 2)	d_1_ = (81 ± 13); 81.2% d_2_ = (831 ± 63); 18.8%
	3	Au@16-3-16/DNA-TC (C_Au@16-3-16_ = 22 nM)	(−62.1 ± 1.0)	d_1_ = (74 ± 11); 93.1% d_2_ = (606 ± 75); 6.9%
	4	Au@16-3-16/DNA-TC (C_Au@16-3-16_ = 40 nM)	(−42.9 ± 0.8)	d_1_ = (70 ± 10); 93.1% d_2_ = (463 ± 67); 6.9%
**Region 2**	5	C_1_ (C_Au@16-3-16_ = 51 nM)	(−36.2 ± 1.6)	d_1_ = (50 ± 10); 99.2% d_2_ = (143 ± 11); 0.8%
	6	Au@16-3-16/DNA-TC (C_Au@16-3-16_ = 62 nM)	(−36.2 ± 1.4)	d_1_ = (52 ± 14); 99.2% d_2_ = (363 ± 15); 0.8%
	7	C_2_ (C_Au@16-3-16_ = 74 nM)	(−35 ± 2)	d_1_ = (61 ± 15); 99.3% d_2_ = (332 ± 21); 0.7%
	8	Au@16-3-16/DNA-TC (C_Au@16-3-16_ = 96 nM)	(−33.3 ± 0.5)	d_1_ = (57 ± 10); 99.2% d_2_ = (455 ± 60); 0.8%
	9	C_3_ (C_Au@16-3-16_ = 130 nM)	(−31.1 ± 0.7)	d_1_ = (68 ± 7); 99.3% d_2_ = (461 ± 54); 0.7%

**Table 2:** MIC values for distinct Au@16-3-16 nanoparticles and Au@16-3-16/DNA-TC (C_i_) complexes. TC control was included for comparative purposes.

System	MIC in *E. coli*	MIC in *S. aureus*
**TC**	5.05 mg/L (25µM)	5.05 mg/L (25 µM)
**N_1_**	25.5 nM *	25.5 nM *
**N_2_**	37.0 nM *	37.0 nM *
**N_3_**	65.0 nM *	16.2 nM *
**C_1_**	5.05 mg/L (25µM)	5.05 mg/L (25 µM)
**C_2_**	5.05 mg/L (25µM)	5.05 mg/L (25 µM)
**C_3_**	5.05 mg/L (25µM)	5.05 mg/L (25 µM)

* Note that the MIC value for TC in N_i_ nanoparticles is equal to zero, given that Ni is prepared in the absence of TC. The MIC values are calculated assuming that N_i_ acts as an antibiotic by itself.

## Data Availability

Data is contained within the article or Supplementary Materials.

## Supplementary Materials

The following supporting information can be downloaded at: https://www.mdpi.com/article/10.3390/pharmaceutics14091941/s1, Table S1. Absorbance of Au@16-3-16 nanoparticles and nanocomplexes over time at maximum SPR. Figure S1: Characterization and chemical structure of the 16-3-16 gemini surfactant compound used for the formation of Au@16-3-16 gold nanoparticles. Figure S2: TEM image and size distribution of Au@16-3-16 nanoparticles. Figure S3: Images of disk diffusion assay for *S. aureus* and *E. coli*. Figure S4: Zeta potential of Au@16-3-16, DNA/TC and C_1_, C_2_ and C_3_ Au@16-3-16/DNA–TC complexes. Figure S5: DLS size distribution by number of Au@16-3-16, DNA/TC and C_1_, C_2_ and C_3_ Au@16-3-16/DNA–TC complexes. Figure S6: Example of microtiter plate with MIC results for *S. aureus*. Figure S7: Example of microtiter plate with MIC results for *E. coli*. Figure S8: Image of the MBC assay for *S. aureus*. Figure S9: Image of the MBC assay for *E. coli*.
