# Supplementary material for: Use of Nanoparticles to Prevent Resistance to Antibiotics—Synthesis and Characterization of Gold Nanosystems Based on Tetracycline
Source: Pharmaceutics. 2022 Sep 14;14(9):1941. doi: 10.3390/pharmaceutics14091941 (PMC9500715; doi:10.3390/pharmaceutics14091941)
Supplement: Supplementary file 1 [file pharmaceutics-14-01941-s001.zip › pharmaceutics-1859075-supplementary.pdf]

pharmaceutics

Supplementary Materials: Use of nanoparticles to prevent resistance to antibiotics—Synthesis and characterization of gold nanosystems based on Tetracycline

Rosa M. Giráldez-Pérez, Elia Grueso, Raquel Jiménez-Aguayo, Alfonso Carbonero, Marina González-Bravo, Edyta Kuliszewska and Rafael Prado-Gotor

NMR Characterization of 16-3-16 gemini surfactant

For NMR spectroscopic measurements, the two compounds (see Fig. S10 for structure) were dissolved in 99.95% $CDCl_3$ (~10 mg in 0.7 mL) and transferred to 5 mm NMR sample tubes (Promochem, Wesel, Germany). Spectra were measured on a Bruker DRX-400 AVANCE spectrometer at 400.13 MHz (^1H) or 100.62 MHz (^{13}C) using the Topspin 1.3 (Bruker, Rheinstetten, Germany). For 1D spectra, 32k data points were recorded and Fourier transformed to spectra with a range of 15 ppm (^1H) and 240 ppm (^{13}C). Two-dimensional COSY, TOCSY, NOESY, HMQC and HMBC spectra were measured with 128 experimental runs having 1024 data points each. Appropriate linear forward prediction, sinusoidal multiplication and Fourier transformation led to 2D-spectra with ranges of 12 ppm and 220 ppm for ^1H and ^{13}C, respectively. Residual $CHCl_3$ was used as the internal standard for ^1H (δ_H 7.24) and $CDCl_3$ for ^{13}C (δ_C 77.0) spectra. Measurement temperature was 298.1 K +/-0.1 K.

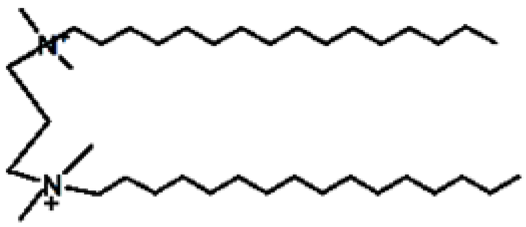

Figure S1. Structure of the 16-3-16 gemini surfactant compound used for the formation of Au@16-3-16 gold nanoparticles.

1,3-Propanediyl-bis-(dimethylhexadecylammonium bromide) (16-3-16). Yield = 68%. Elemental analysis ($C_{39}H_{84}N_2Br_2$, M_r=740.9057). Calc. for trihydrate (%): C 63.22; H 11.43; N 3.78. Found (%): C 61.78; H 10.98; N 3.67. ^{1}H-NMR (400 MHz, $CDCl_3$, δ in ppm): 3.87 (m, 4H, N^+-C*H*$_2$-CH_2-C*H*$_2$-N^+); 3.49 (m, 4H, N^+-C*H*$_2$-); 3.36 (s, 12H, N^+-C*H*$_3$); 2.74 (m, 2H, N^+-CH_2-C*H*$_2$-CH_2-N^+); 1.79 (m, 4H, N^+-CH_2-C*H*$_2$-); 1.38-1.24 (m, 52H, -(C*H*$_2$)$_{13}$-); 0.87 (t, 6H, -C*H*$_3$). ^{13}C NMR (100 MHz, $CDCl_3$, δ in ppm): 67.3 (N^+-CH_2-); 61.6 (N^+-*C*H_2-CH_2-*C*H_2-N^+); 51.7 (N^+-*C*H_3); 32.2, 29.9, 29.8, 29.8, 29.8, 29.8, 29.6, 29.6, 29.6, 29.5, 29.5, 26.7, 23.1 (-(*C*H_2)$_{13}$-); 23.4 (N^+-CH_2-*C*H_2-); 19.2 (N^+-CH_2-*C*H_2-CH_2-N^+); 14.6 (-*C*H_3). Mass spectroscopy: (m/z) = 740.50; $[M^{++\,79}Br^-]^+$ = 661.5; $[M^{++\,81}Br^-]^+$ = 659.4; [(M - 2Br) / 2]$^+$ = 289.7.

Table S1. Stability of the Au@16-3-16 and Au@16-3-16/DNA-TC (C_i) nanosystems over time after preparation of different nanoformulations. Absorbance measurements were carried out at the maximum SPR band of gold nanosystems in each case.

**Nanosystem**	**Au@16-3-16**	**C_1**	**C_2**	**C_3**
In situ $\lambda_{MAX,SPR}$/$Abs_{MAX,SPR}$	519 nm/0.0818	521 nm/0.3020	521 nm/0.3852	522 nm/0.6289
24 hours $\lambda_{MAX,SPR}$/$Abs_{MAX,SPR}$	519 nm/0.0818	521 nm/0.2661	521 nm/0.3712	522 nm/0.4878
48 hours $\lambda_{MAX,SPR}$/$Abs_{MAX,SPR}$	519 nm/0.0819	521 nm/0.2623	521 nm/0.3577	522 nm/0.4686
1 week $\lambda_{MAX,SPR}$/$Abs_{MAX,SPR}$	519 nm/0.0820	521 nm/0.2677	521 nm/0.3738	522 nm/0.4494
2 weeks $\lambda_{MAX,SPR}$/$Abs_{MAX,SPR}$	519 nm/0.0833	521 nm/0.2635	521 nm/0.3700	522 nm/0.4063
3 weeks $\lambda_{MAX,SPR}$/$Abs_{MAX,SPR}$	519 nm/0.0841	521 nm/0.2663	521 nm/0.3572	522 nm/0.4675
1 month $\lambda_{MAX,SPR}$/$Abs_{MAX,SPR}$	519 nm/0.0874	521 nm/0.2554	521 nm/0.3477	522 nm/0.4732

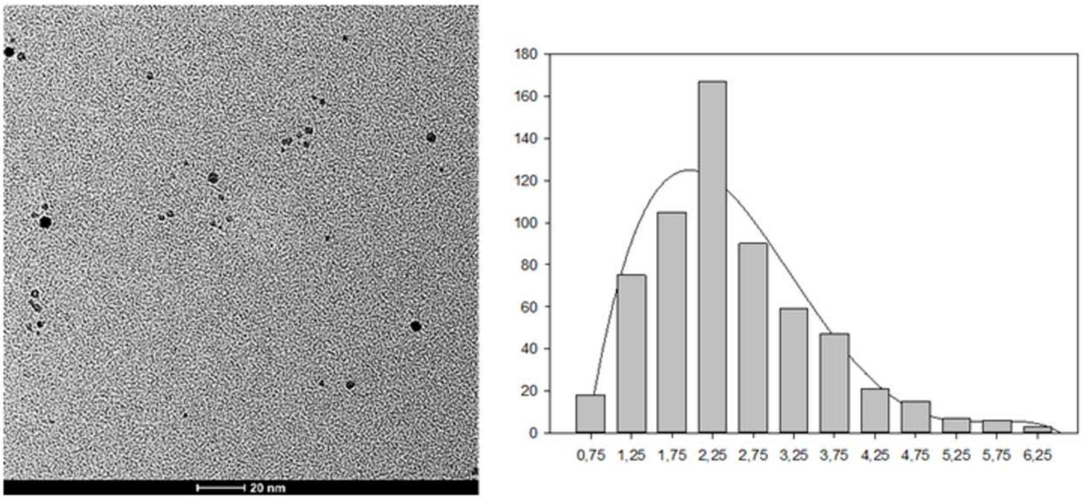

Figure S2. Microphotograph made with TEM TALOS, showing the gold core of the nanoparticles and the corresponding histogram of the size distribution made with the Image J program on 500 gold cores of the nanoparticles.

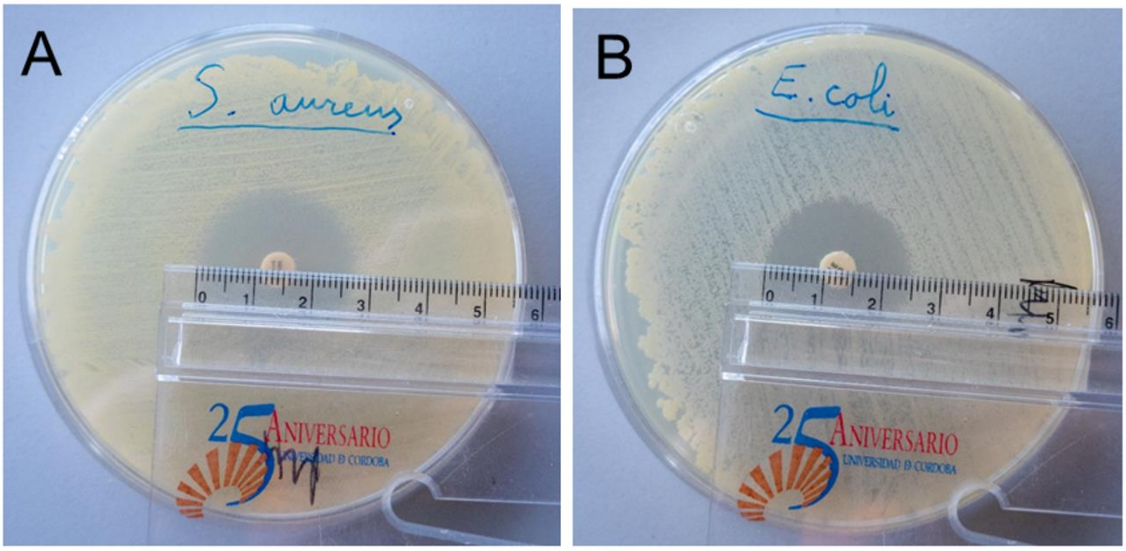

Figure S3. Disk diffusion test of reference *S. aureus* (A) and *E. coli* (B) strains against tetracycline.

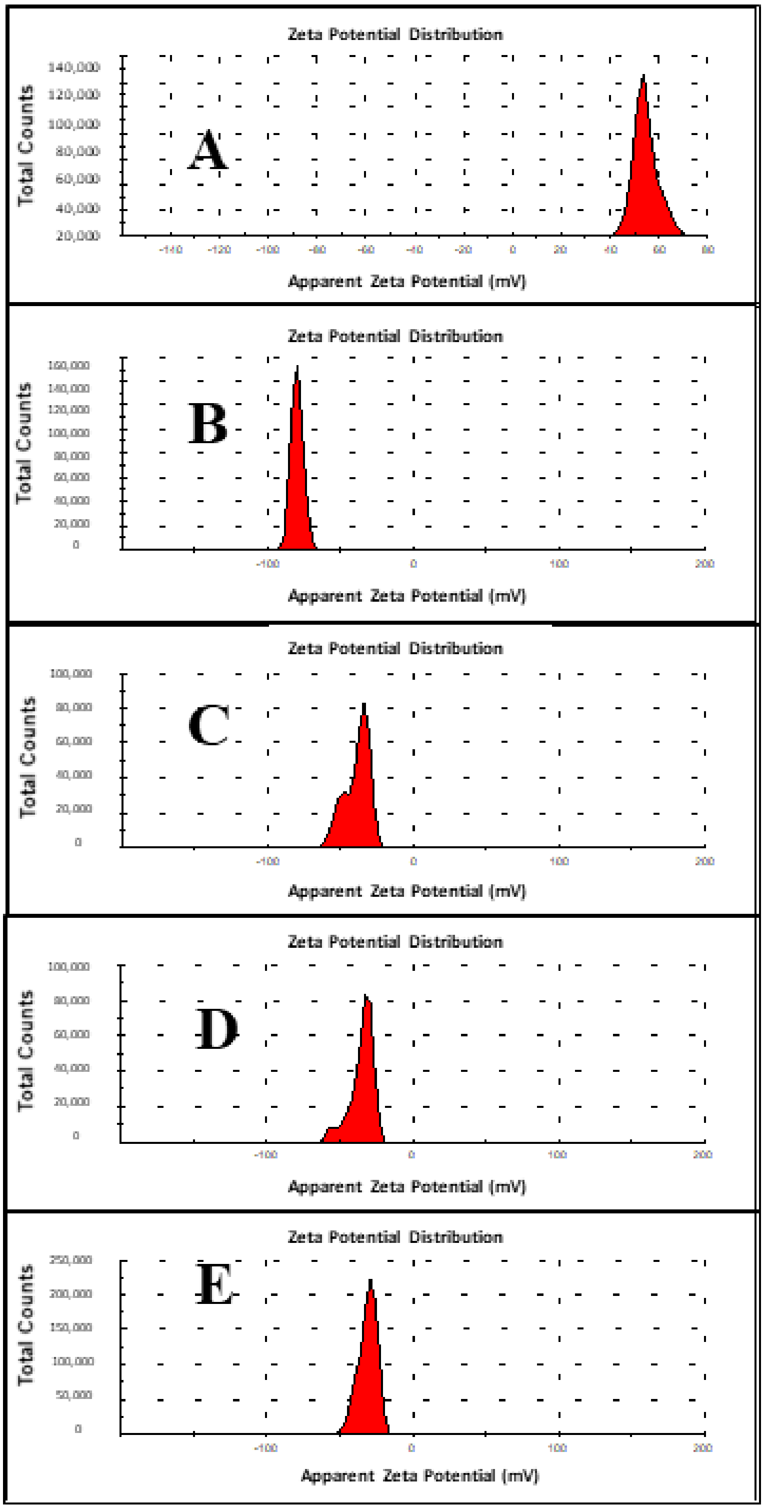

Figure S4. Zeta potential of Au@16-3-16, DNA/TC complex and Au@16-3-16 /DNA-TC nanosystems in water. (**A**) Au@16-3-16, $C_{Au@16\text{-}3\text{-}16}$ = 17.0 nM (**B**) DNA/TC complex, $C_{Au@16\text{-}3\text{-}16}$ = 0 nM (**C**) C_1, $C_{Au@16\text{-}3\text{-}16}$ = 51 nM; (**D**) C_2, $C_{Au@16\text{-}3\text{-}16}$ = 74 nM, and (**E**) C_3, $C_{Au@16\text{-}3\text{-}16}$ = 130 nM. A fixed concentration of DNA and TC (C_{DNA} = 100 μM and C_{TC} = 50 μM) were used for the preparation of the complexes.

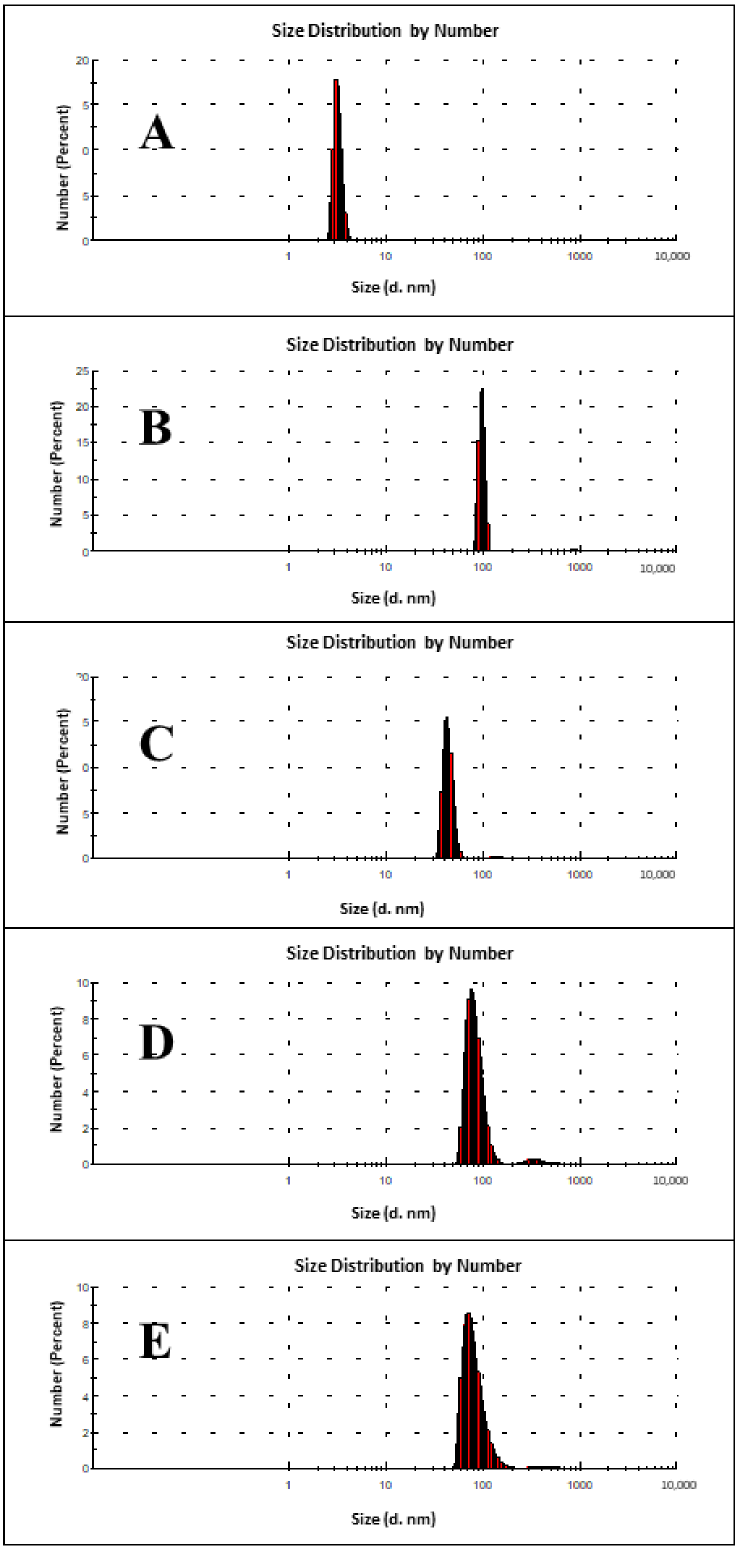

Figure S5. DLS size distribution by number of Au@16-3-16, DNA/TC complex and Au@16-3-16 /DNA-TC nanosystems in water. (**A**) Au@16-3-16, $C_{Au@16\text{-}3\text{-}16}$ = 17.0 nM (**B**) DNA/TC complex, $C_{Au@16\text{-}3\text{-}16}$ = 0 nM (**C**) C_1, $C_{Au@16\text{-}3\text{-}16}$ = 51 nM; (**D**) C_2, $C_{Au@16\text{-}3\text{-}16}$ = 74 nM, and (**E**) C_3, $C_{Au@16\text{-}3\text{-}16}$ = 130 nM. A fixed concentration of DNA and TC (C_{DNA} = 100 µM and C_{TC} = 50 µM) were used for the preparation of the complexes.

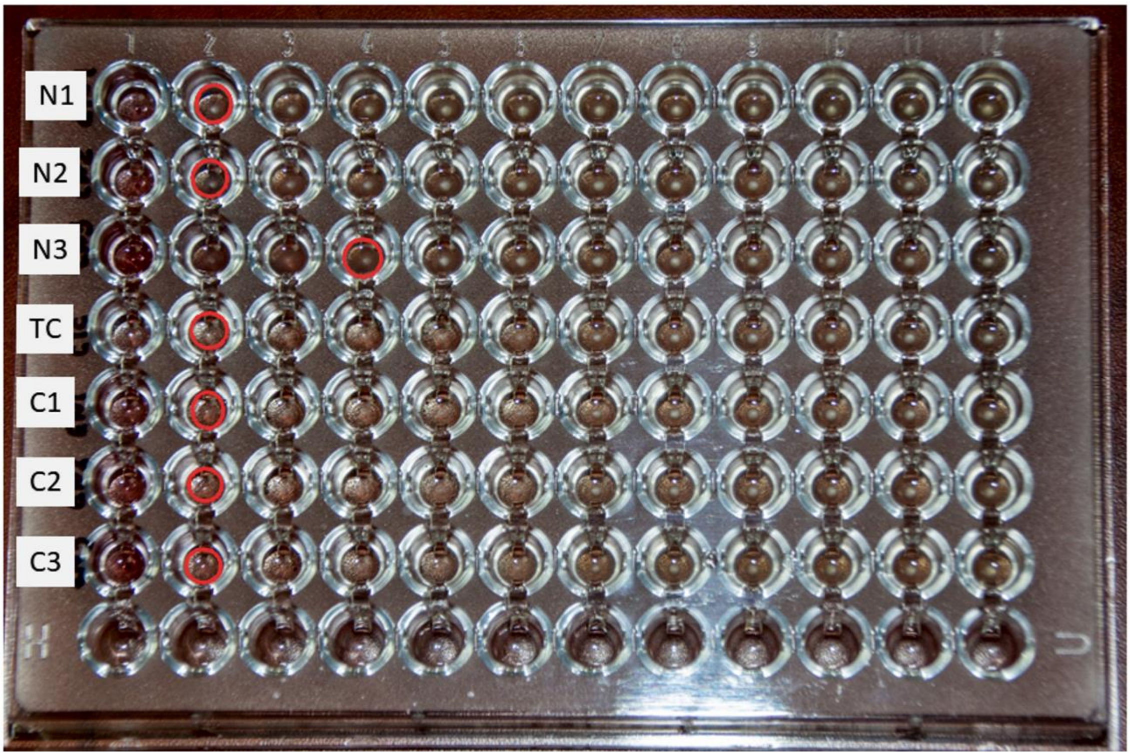

Figure S6. Example of microtiter plate with MIC results for *S. aureus*. The red circles denote the presence of the first bacterial button in each case.

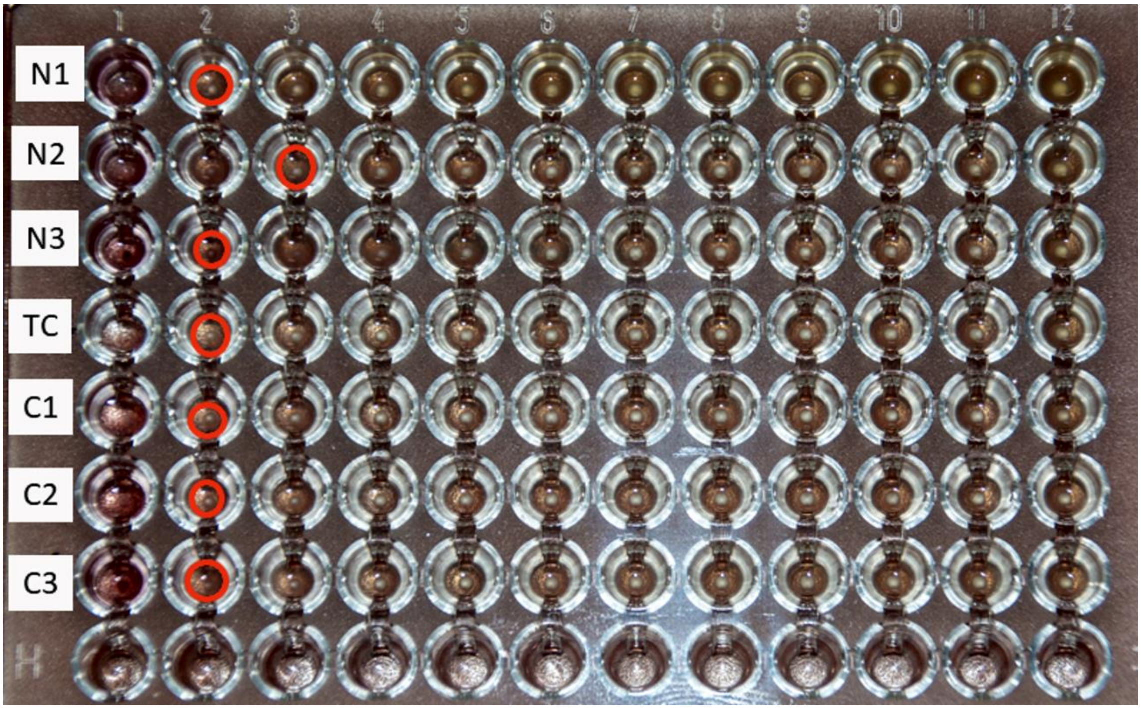

Figure S7. Example of microtiter plate with MIC results for *E. coli.* The red circles denote the presence of the first bacterial button in each case.

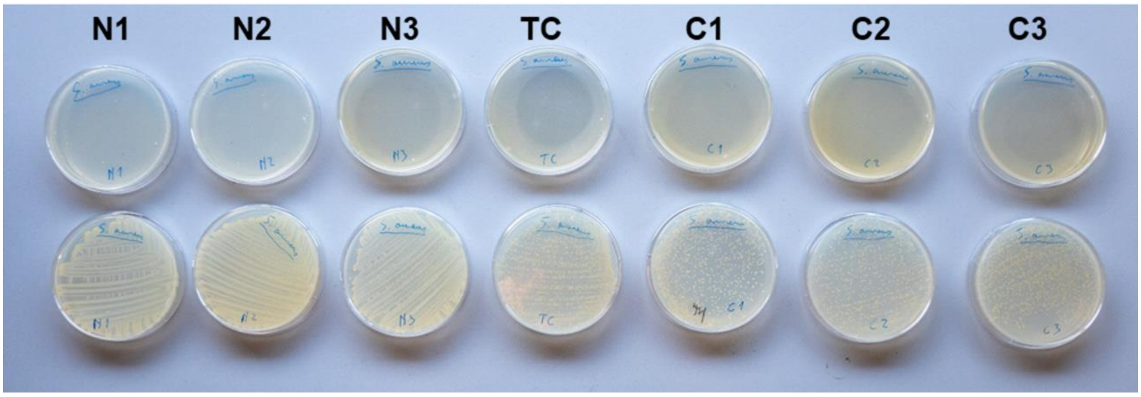

Figure S8. MBC of *S. aureus*. The first row corresponds to not diluted nanosystems and controls, and the second to the first dilution. The rest of the dilutions showed a dense growth. N_1, N_2 and N_3 formulations correspond to $C_{Au@16\text{-}3\text{-}16}$ = 51 nM, 74 nM and 130 nM, respectively. TC control (C_{TC} = 50 μM) and C_1, C_2, and C_3 compacted Au@16-3-16/DNA-TC nanosystems.

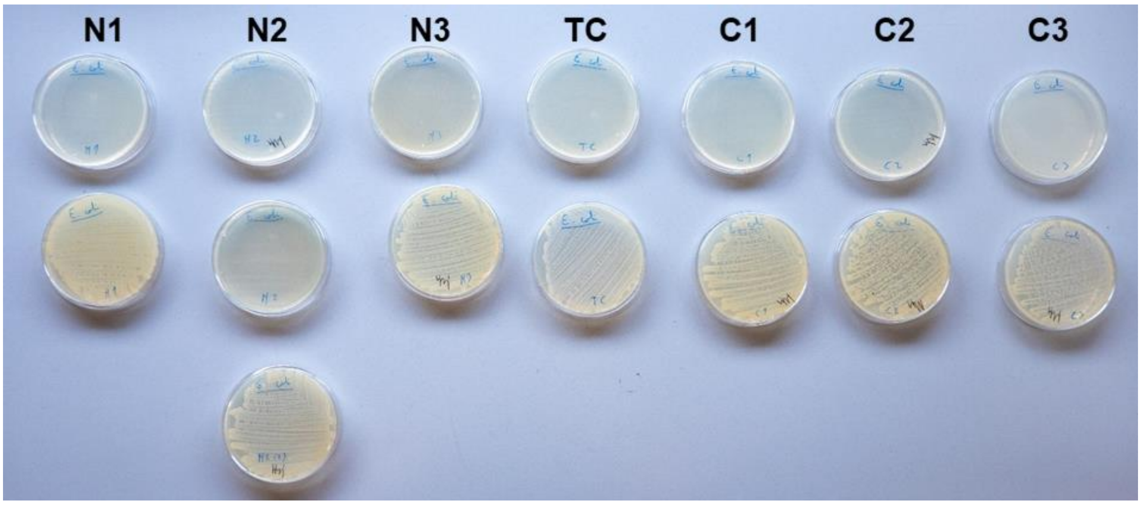

Figure S9. MBC of *E. coli.* The first row corresponds to not diluted nanosystems and controls, and the second and third to the first and second dilution, respectively. Control nanoparticle N_2 did not exhibit bacterial growth until the second dilution, as shown in the figure. The rest of the dilutions showed a dense growth. N_1, N_2 and N_3 formulations correspond to $C_{Au@16\text{-}3\text{-}16}$ = 51 nM, 74 nM and 130 nM, respectively. TC control (C_{TC} = 50 μM) and C_1, C_2, and C_3 Au@16-3-16/DNA-TC compacted nanosystems.
